# Hybrid zone of a tree in a Cerrado/Atlantic Forest ecotone as a hotspot of genetic diversity and conservation

**DOI:** 10.1002/ece3.8540

**Published:** 2022-01-22

**Authors:** André Carneiro Muniz, Ricardo José Gonzaga Pimenta, Mariana Vargas Cruz, Jacqueline Gomes Rodrigues, Renata Santiago de Oliveira Buzatti, Myriam Heuertz, José P. Lemos‐Filho, Maria Bernadete Lovato

**Affiliations:** ^1^ Departamento de Genética, Ecologia e Evolução Universidade Federal de Minas Gerais Belo Horizonte Brazil; ^2^ Biogeco INRAE, Univ. Bordeaux Cestas France; ^3^ Departamento de Botânica Universidade Federal de Minas Gerais Belo Horizonte Brazil

**Keywords:** conservation, ecotypes, hybrid zone, Neotropical tree, *Plathymenia*, savanna–forest ecotone

## Abstract

The Cerrado, the largest Neotropical savanna, and the Brazilian Atlantic Forest form large ecotonal areas where savanna and forest habitats occupy adjacent patches with closely related species occurring side by side, providing opportunities for hybridization. Here, we investigated the evolutionary divergence between the savanna and forest ecotypes of the widely distributed tree *Plathymenia reticulata* (*n* = 233 individuals). Genetic structure analysis of *P*. *reticulata* was congruent with the recognition of two ecotypes, whose divergence captured the largest proportion of genetic variance in the data (*F*_CT_ = 0.222 and *F*_ST_ = 0.307). The ecotonal areas between the Cerrado and the Atlantic Forest constitute a hybrid zone in which a diversity of hybrid classes was observed, most of them corresponding to second‐generation hybrids (F2) or backcrosses. Gene flow occurred mainly toward the forest ecotype. The genetic structure was congruent with isolation by environment, and environmental correlates of divergence were identified. The observed pattern of high genetic divergence between ecotypes may reflect an incipient speciation process in *P*. *reticulata*. The low genetic diversity of the *P*. *reticulata* forest ecotype indicate that it is threatened in areas with high habitat loss on Atlantic Forest. In addition, the high divergence from the savanna ecotype suggests it should be treated as a different unit of management. The high genetic diversity found in the ecotonal hybrid zone supports the view of ecotones as important areas for the origin and conservation of biodiversity in the Neotropics.

## Graphical abstract

Here, we investigated the evolutionary divergence between the savanna and forest ecotypes of the tree *Plathymenia reticulata* in the Cerrado, the largest Neotropical savanna, and the Brazilian Atlantic Forest. We found that the ecotonal areas between the Cerrado and the Atlantic Forest constitute a hybrid zone in which a diversity of hybrid classes were observed, most of them corresponding to second generation hybrids. The high genetic diversity found in the ecotonal hybrid zone supports the view of ecotones as important areas for the origin and conservation of biodiversity in the Neotropics.

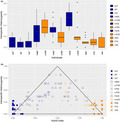

## 1 INTRODUCTION

Understanding the processes that underlie genetic divergence between lineages is one of the major goals of evolutionary biology. The speciation process occurs along a continuum of genetic divergence where the incipient lineages often undergo a period of partial reproductive isolation until they become fully isolated (Christie & Strauss, 2018; Dufresnes et al., 2019; Kopp & Frank, 2005; Wu, 2001). It is frequently recognized that most genetic divergence during speciation occurs owing to allopatric divergence, where there is little or no gene flow between geographically isolated populations (Coyne, 1992). However, a growing body of evidence suggests that speciation with gene flow is more common in nature than previously thought (Choi et al., 2019; Feder et al., 2012; Hanson et al., 2016; Rifkin et al., 2019). The genomic divergence under speciation with gene flow can be very different from that of allopatric divergence because gene flow can homogenize the genomic background, while islands of divergence may result for loci under divergent natural selection (Feder et al., 2012). In speciation with gene flow models, divergence between incipient lineages is mainly driven by two processes, divergent selection that favors the divergence of groups of individuals with extreme traits, and nonrandom or assortative mating (Servedio, 2016).

Differential selection and nonrandom mating may lead to an isolation by environment (IBE) pattern, in which the association between genetic and environmental distance is stronger than that between genetic and spatial distance between populations (Wang & Bradburd, 2014). IBE is agnostic to the cause of divergence and rather a description of the pattern since many processes such as selection against immigrants, reduced hybrid fitness, and biased effective dispersal can generate a pattern of IBE (Wang & Bradburd, 2014). Under IBE, even neutral markers are expected to show some level of correlation with the environmental distance due to the impact of differential gene flow among ecologically similar or dissimilar habitats (Nosil et al., 2008, 2009). Disentangling IBE from isolation by distance (IBD), where lineages diverge due to limited dispersal of individuals, may be statistically difficult since the environmental distance and the spatial distance are often correlated (Nadeau et al., 2016). However, evaluating the relative importance of IBE versus IBD in the genetic divergence can give some insights into the possible causes of evolutionary divergence of lineages.

In the speciation process, some degree of hybridization may be commonly found. Hybridization, estimated to occur in 25% of plant taxa (Mallet, 2005), can create new gene combinations in later generation hybrids, lead to new species through hybrid speciation (Abbott, 2017; Abbott et al., 2013; Maguilla & Escudero, 2016; Paun et al., 2009), and influence the patterns of genetic variability of species through genomic introgression (Francisco et al., 2018; Gompert et al., 2017; Rieseberg & Carney, 1998). Furthermore, hybridization can lead to the disruption of reproductive barriers between incipient lineages reverting the speciation process (Abbott et al., 2013; Soltis & Soltis, 2009) or can lead to extinction through genetic or demographic swamping (Rhymer & Simberloff, 1996; Todesco et al., 2016). Therefore, the study of gene flow between lineages and the genotypic composition of hybrid zones can help us investigate evolutionary questions related to the formation of species and the development of reproductive barriers between lineages.

Neotropical ecosystems harbor high levels of biodiversity and species endemism, but the causes of such remarkable diversification are still poorly understood. The Brazilian Atlantic Forest, constituted by evergreen and semideciduous forest (Oliveira‐Filho & Fontes, 2000), and the Cerrado, considered to be the most species‐rich savanna of the world, are recognized hotspots for conservation (Myers et al., 2000). The Cerrado features poorer and more acid soils and its climate is characterized by a long dry season with less annual precipitation in comparison with the Atlantic Forest (Eiten, 1972; Klink & Machado, 2005). In contrast with the Atlantic Forest, natural fires are common in the Cerrado (Hoffmann et al., 2003).

The Cerrado and Atlantic Forest are in contact in southeastern Brazil where they form large ecotonal areas where savanna and forest habitats occur in adjacent patches with differences in tree density and species composition (Durigan & Ratter, 2006). In these ecotonal areas, pairs of closely related species from the Cerrado and Atlantic Forest are found side by side (Hoffmann & Franco, 2003; Hoffmann et al., 2003). This spatial proximity can provide opportunities for gene flow between closely related species if reproductive isolation between these taxa is not completed. In fact, a few studies using nuclear markers with sampling in contact zones between the Atlantic Forest and the Cerrado have found gene flow between closely related tree taxa (Cavallari et al., 2010; Lacerda et al., 2002; Muniz et al., 2020; Resende‐Moreira et al., 2017).

*Plathymenia reticulata* Benth (Fabaceae, Caesalpinioideae – LPWG, 2017) is the single species of the *Plathymenia* genus (Warwick & Lewis, 2003) and features a widespread distribution in both biomes, the Cerrado and the Atlantic Forest. The species presents two ecotypes, one associated with forest habitats and the other with savanna habitats (Lemos‐Filho et al., 2008). The *P*. *reticulata* forest ecotype usually shows straight trunks reaching up to 30 m in height and up to 70 cm in diameter, whereas the savanna ecotype reaches 6–12 m in height and 30–50 cm in trunk diameter and displays a shorter and tortuously branched trunk (Lorenzi, 1992, Figure S1). The ecotypes also show differences in ecophysiological traits, which are associated to adaptive responses between savanna and forest habitats (Lemos‐Filho et al., 2008), such as in vegetative phenology, seed and fruit morphology, seed dormancy and germination, phenotypic plasticity in response to light availability, and stem radial growth (Goulart, Lemos‐Filho, & Lovato, 2005, 2006; Goulart et al., 2011; Toledo et al., 2012). In addition, these studies showed that the populations occurring in the ecotone between the Atlantic Forest and the Cerrado are generally morphophysiologically intermediate relative to populations from the Atlantic Forest and the Cerrado.

A common garden experiment with *P*. *reticulata* progenies of populations from the Cerrado, the Atlantic Forest, and ecotonal areas showed that individuals developed the morphological and physiological characteristics of their respective habitats (Goulart et al., 2011), indicating that their differences are genetically determined. Genetic studies of *Plathymenia* showed admixture in an ecotonal population suggesting gene flow between the two ecotypes (Lacerda et al., 2002) and a pattern of plastid DNA variation congruent with a diverse, persistent central‐north Cerrado population as a potential source of population expansion after the Last Glacial Maximum (Novaes et al., 2010). Therefore, *P*. *reticulata* likely shows a complex pattern of population genetic structure influenced by range dynamics due to Quaternary climate oscillations, the divergence of ecotypes, and admixture in areas where both ecotypes come into contact.

In this study, we sampled *P*. *reticulata* in the Cerrado and the Atlantic Forest, and in ecotonal areas between the two biomes. We used nuclear microsatellite markers to investigate the patterns and the putative drivers of genetic divergence between savanna and forest ecotypes and to characterize a putative hybrid zone in the ecotonal areas. Specifically, we addressed the following questions: (1) What level of genetic diversity characterizes *P*. *reticulata* and how is genetic diversity structured geographically, between and within ecotypes? (2) How much gene flow occurs in ecotonal populations, and is gene flow asymmetric between ecotypes? (3) What is the genotypic composition of the hybrid populations, are they mostly F1s or later generation hybrids? (4) What is the effect of environmental variation on the genetic structure? We discuss the evolutionary consequences of divergence in *P*. *reticulata* and the implications for conservation of genetic diversity of the ecotonal populations.

## 2 MATERIALS AND METHODS

### 2.1 Sampling strategy

**FIGURE 1:**
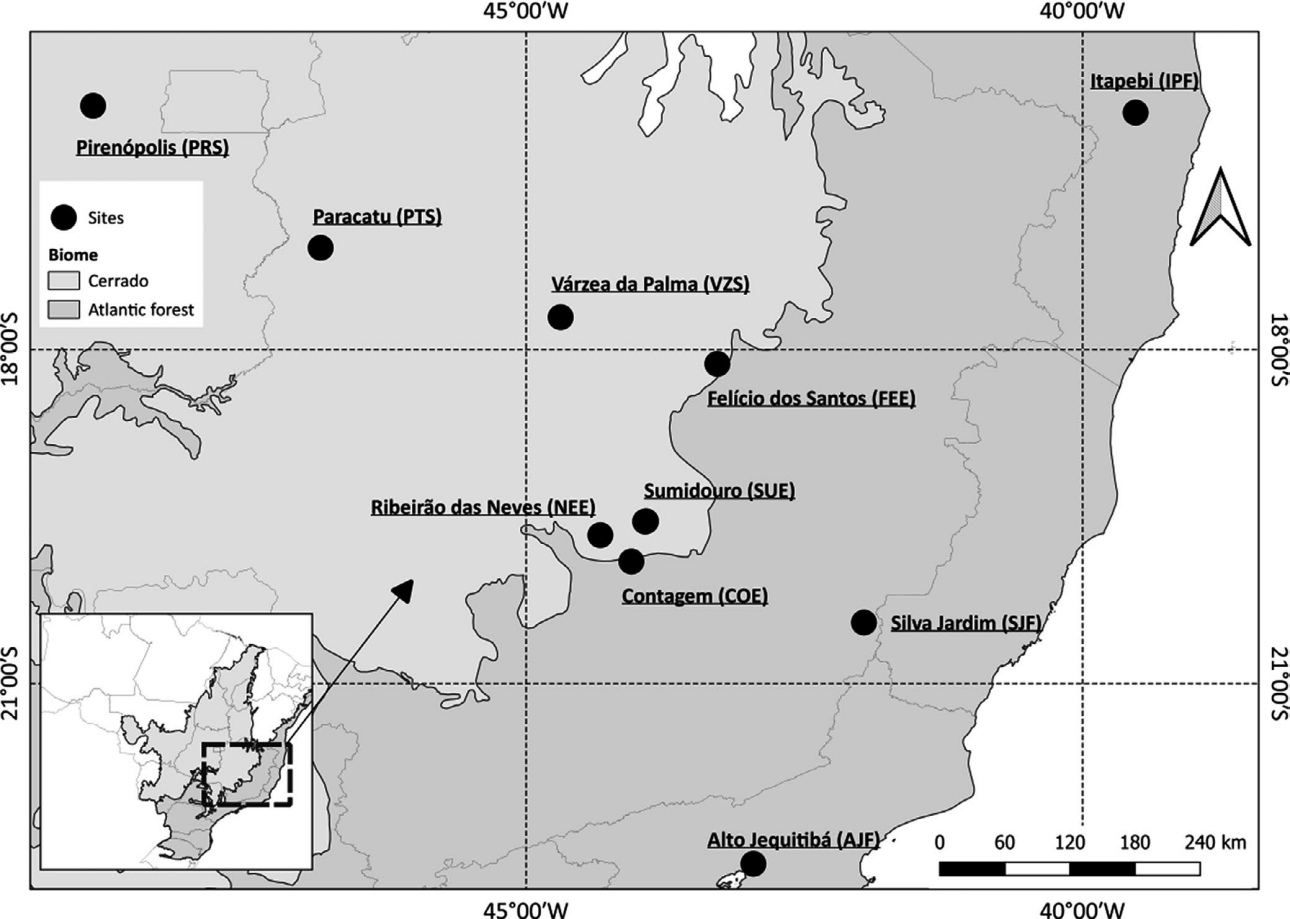
Map showing the sampling localities of *Plathymenia reticulata* in the Brazilian Atlantic Forest (dark gray) and the Cerrado (light gray) biomes, and in the ecotone between the two

We sampled 233 individuals of *P*. *reticulata*, comprising the savanna and forest ecotypes in 10 localities from three different habitats with three localities sampled in the Atlantic Forest (SJF, AJF, and IPF) and the Cerrado (PRS, PTS, and VZS), and four localities sampled in the ecotone between the two biomes (NEE, COE, SUE, and FEE) (Figure 1). In the SUE and FEE localities of the ecotone, both savanna and forest ecotypes occur in sympatry/parapatry, whereas in COE and NEE, only the savanna or forest ecotypes were found, respectively. In the ecotonal localities, we used the letters s (savanna) or f (forest) in the locality code to identify the ecotype we were referring to. For example, in the SUE locality, we sampled s‐SUE and f‐SUE populations of each ecotype. The classification of individuals in ecotypes was based on differences in size (savanna individuals did not exceed 10 m in height of the canopy, whereas forest individuals measured from 15 to 30 m in height), trunk (savanna individuals had a tortuous and twisted trunk, whereas forest individuals had a straight trunk), and bark (which was more suberous in the savanna ecotype) (Figure S1).

### 2.2 DNA isolation, amplification, and genotyping

Leaf or bark tissue samples were dried on silica gel and kept frozen at −20°C. Genomic DNA was extracted from leaves using the sorbitol buffer‐based method according to the protocol described by Souza et al. (2012). For bark tissue, we used the protocol developed by Novaes et al. (2009). We genotyped individuals for 11 microsatellite markers previously developed for *P*. *reticulata*: Pre05, Pre10, Pre11, Pre14, Pre15, Pre16, and Pre23 (Cruz et al., 2012) and Pr18, Pr30, Pr71, and Pr80 (Oliveira et al., 2012). Polymerase chain reactions (PCRs) were performed following the conditions established by Oliveira et al. (2012). Genotyping was performed using the automated sequencers MegaBACE 1000 (GE Healthcare, Chicago, Illinois, EUA) or ABI 3730xl DNA Analyzer (Applied Biosystems, Foster City, California, EUA) through capillary electrophoresis. Allele size was determined by comparison with either GeneScan ROX‐500 or LIZ‐500 Size Standards (Applied Biosystems) using MegaBACE Fragment Profiler version 1.2 (GE Healthcare) and Geneious version 7.1.3 (Biomatters Ltd., Auckland, New Zealand). The presence of null alleles at each locus was examined by ecotype by population with 1000 Monte Carlo simulations using Micro‐Checker version 2.3.3 (Van Oosterhout et al., 2004) and the linkage equilibrium among pairs of loci was evaluated using FSTAT version 2.9.3.2 (Goudet, 2002). Micro‐Checker analysis found significant evidence for the presence of null alleles in all *P*. *reticulata* localities, except for SJF and s‐SUE (Table S1). However, the loci did not show significant null alleles regularly across localities, which can indicate that these loci are not in Hardy–Weinberg equilibrium in these populations; therefore, all subsequent analyses were performed with the full, noncorrected dataset. No significant linkage disequilibrium was found among loci in any pairwise comparison at the population level.

### 2.3 Genetic diversity and structure of *Plathymenia reticulata*, and gene flow between ecotypes

Arlequin version 3.5 (Excoffier & Lischer, 2010) was used to estimate the mean number of alleles per locus (*A*) and the mean observed (*H*_O_) and expected (*H*_E_) heterozygosities. The allelic richness (*A*_R_) and the inbreeding coefficient (*F*_IS_) were estimated in FSTAT version 2.9.3.2 (Goudet, 2002). The hierarchical partitioning of genetic variance was investigated using the standard analysis of molecular variance (AMOVA) according to the method of Weir and Cockerham (1984), implemented in Arlequin version 3.5 (Excoffier & Lischer, 2010). We considered variation between ecotypes as the highest hierarchical level of genetic divergence, followed by variation among populations within ecotypes and variation within populations, and computed the corresponding *F* statistics. The pairwise genetic differentiation between localities was evaluated by *F*_ST_ estimated in Arlequin version 3.5 (Excoffier & Lischer, 2010).

We used POPTREEW (Takezaki et al., 2014) to build a tree from allele frequency data using the neighbor‐joining (NJ) method (Saitou & Nei, 1987) based on Nei's standard genetic distance (*D*_ST_) (Nei, 1972) with sample size bias correction. We used the *P*. *reticulata* populations without the admixed individuals in order to evaluate the phylogenetic relationships among populations as well as between ecotypes. Bootstrap tests (Felsenstein, 1985) were performed for the evaluation of confidence intervals of the tree's branches in order to estimate the robustness of the tree.

We investigated the genetic structure as well as estimated admixture in *P*. *reticulata* using the following algorithms: (1) discriminant analysis of principal components (DAPC) implemented in the *adegenet* package version 2.1.4 in R 4.1.0 (Jombart, 2008; Jombart et al., 2010; R Core Team, 2020) and (2) the Bayesian clustering method implemented in STRUCTURE version 2.3.4 (Hubisz et al., 2009; Pritchard et al., 2000). DAPC has the advantage of not relying on assumptions of Hardy–Weinberg equilibrium or linkage equilibrium. In the DAPC, we used the sequential *k‐means* clustering method implemented in the *adegenet* package (Jombart et al., 2010) to choose the number of genetic groups that best explains the genetic variability in *P*. *reticulata* and then used these groups in DAPC to estimate the posterior assignment probability of individuals to groups. After the evaluation of the population structure based on the two ecotypes, we applied the DAPC algorithm in each ecotype separately using only the individuals that showed a posterior assignment probability higher than 0.85, which we considered to be pure individuals. In STRUCTURE version 2.3.4, we set the number of clusters from *K* = 1 to *K* = 13 and ran 10 independent iterations per *K* assuming an admixture model with correlated allelic frequencies between localities. We ran 100,000 steps as burn‐in followed by 1,000,000 Markov chain Monte Carlo (MCMC) iterations. The most probable number of genetic clusters was determined by the ad hoc statistic Δ*K* (Evanno et al., 2005), computed in Structure Harvester version 0.6.94 (Earl & vonHoldt, 2012). Besides the Evanno's Δ*K* method, we also used “MedMeaK” (median of means), “MaxMeaK” (maximum of means), “MedMedK” (median of medians), and “MaxMedK” (maximum of medians) statistics in StructureSelector (Li & Liu, 2018) based on the method of Puechmaille (2016). In this method, a subpopulation is assigned to a cluster if its arithmetic mean (for MedMeaK and MaxMeaK) or its median (for MedMedK and MaxMedK) membership coefficient to that cluster is greater than a threshold value (set to 0.5), therefore ensuring that a subpopulation cannot belong to more than one cluster. We averaged the admixture coefficients using CLUMPP version 1.1.2 (Jakobsson & Rosenberg, 2007) and plotted the values using the ggplot2 package version 3.3.5 in the R software (Wickam, 2009). We also tested for genetic structure within each ecotype, using all individuals identified as pure (<15% or >85% ancestry threshold) in the STRUCTURE analysis of the full dataset. For each ecotype, we ran a separated analysis setting *K* = 1 to *K* = 7.

To estimate rates of recent migration between the two ecotypes (based on phenotype classification), we used BayesAss 3.0 (Wilson & Rannala, 2003). This software uses a Bayesian framework and MCMC iterations to estimate the posterior mean of recent migration rates (mainly the last two generations) and the standard deviation of the marginal posterior distribution from multilocus genotypes for a group of populations in a sample. The software provides estimates using the gametic disequilibrium among immigrants and their descendants. This method relaxes the assumption of previous Hardy–Weinberg equilibrium within populations to assign individuals to populations and identify migrants. We estimated the migration rates between ecotypes in the following datasets: (1) the whole sample of individuals of each ecotype, (2) the ecotonal individuals of the two ecotypes, (3) FEE locality, (4) SUE locality, and (5) forest ecotype of NEE and savanna ecotype of COE, as each of these localities has only one ecotype, but they are spatially close to each other.

### 2.4 Genotypic composition of *Plathymenia* hybrids in the ecotone

We investigated the genotypic class composition of *P*. *reticulata* individuals using NewHybrids version 1.1 (Anderson & Thompson, 2002). The implemented method calculates the posterior probability of individuals to belong to predetermined genotypic classes (Anderson & Thompson, 2002). We applied the method using two different sets of genotypic classes. First, we used the six default predetermined genotypic classes of NewHybrids, two purebred parental classes P1 (savanna) and P2 (forest), F1 (savanna × forest) and F2 (F1 × F1) hybrids, and one backcross of F1 with each parental class (B1 = F1 × savanna and B2 = F1 × forest). Secondly, we investigated more advanced generation hybrids using the following additional six classes of backcrosses: B3 = F2 × savanna and B4 = F2 × forest, B5 = savanna × (F1 × savanna) and B6 = forest × (F1 × forest), B7 = savanna × (F2 × savanna) and B8 = forest × (F2 × forest), according to the genotypic classes in Chhatre et al. (2018) and Buck et al. (2020) (Table S2). In both analyses, we ran 1,000,000 MCMC iterations with a burn‐in period of 100,000 iterations using Jeffrey's‐like priors for mixing proportions and allele frequencies and assuming equal prior probabilities for all genotypic classes.

We further investigated the genotypic composition of *P*. *reticulata* individuals using the hybrid index estimated in the *introgress* package version 1.2.3 in R (Gompert & Buerkle, 2010). The computation of the hybrid index is based on the maximum likelihood method and estimates the genetic contribution of hybridizing “parental” populations to individuals of unknown ancestry (Buerkle, 2005). Here, we used as pure parental individuals those from the Atlantic Forest and Cerrado localities, respectively, to estimate the hybrid index of those individuals from the ecotone localities. In addition, we calculated the interspecific (interecotypic) heterozygosity (*Q*_12_), which is the fraction of loci that combine the ancestry of the two parental taxa (Fitzpatrick & Irwin, 2012). The combination of *Q*_12_ with the hybrid index can give insights into the history of hybridizing taxa through the summary of the genomic composition of individuals (Gompert & Buerkle, 2016). For example, an F2 individual may show a hybrid index of 0.5 just like F1s. However, F1s show *Q*_12_ = 1.0, whereas F2 and later generation hybrids have lower values of *Q*_12_ owing to the breakup of linkage disequilibria because of genome reshuffling in later generations of hybridization.

### 2.5 Evaluation of hybrid assignment in the NewHybrids

The efficiency of NewHybrids to identify and classify hybrids was evaluated using purebred parental and hybrid individuals simulated using the function *hybridize* in the *adegenet* package (Jombart, 2008). We simulated five datasets based on the allele frequencies of pure (*Q* higher than 0.85 or lower than 0.15) *P*. *reticulata* individuals of the Atlantic Forest and the Cerrado areas, respectively. The simulated datasets comprised 100 individuals of each parental and 100 individuals of each hybrid class, F1, F2, and backcrosses of F1 with each parental (B1 and B2). We also simulated more advanced generation hybrids including F3 (F2 × F2) and F4 (F3 × F3), B3 = F2 × savanna and B4 = F2 × forest, B5 = savanna × (F1 × savanna) and B6 = forest × (F1 × forest), B7 = savanna × (F2 × savanna) and B8 = forest × (F2 × forest), based on Chhatre et al. (2018) and Buck et al. (2020) (Table S2). To evaluate the performance of NewHybrids to distinguish hybrid classes, we ran the simulated dataset of six genotypic classes using six genotypic classes in NewHybrids, the 14 simulated genotypic classes using six genotypic classes in NewHybrids, and the 14 simulated genotypic classes using 12 genotypic classes in NewHybrids (Table S2). Finally, we set four thresholds for posterior probabilities of assignment to determine genotypic classes (0.5, 0.75, 0.85, and 0.9) to evaluate the proportion of correct and incorrect assignments. We also assessed the efficiency of STRUCTURE and *introgress* to detect and classify hybrids based on simulated data (Appendix S1).

### 2.6 Drivers of divergence of *Plathymenia reticulata* ecotypes

We examined the effect of edaphic and bioclimatic variables on genetic differentiation between *P*. *reticulata* populations using two approaches: (1) Mantel tests as a first approach to assess effects of isolation by distance and isolation by environment and (2) distance‐based redundancy analysis, dbRDA. For Mantel tests, we calculated the pairwise spatial distance between populations using the coordinates of the localities separated by ecotypes with the *geodist package* version 0.0.7 in R (Padgham & Sumner, 2021). Thus, we had six ecotonal populations since the populations of SUE and FEE were separated in f‐SUE and s‐SUE and in f‐FEE and s‐FEE, respectively. As environmental predictors, we used the edaphic variables *bulk density* of the fine earth fraction (BDOD), *soil* pH (pH), *soil organic carbon* (SOC), and *cation exchange capacity* of the soil (CEC), which were downloaded from https://files.isric.org/soilgrids/latest/data/ and the 19 bioclimatic variables from the worldclim2 dataset https://www.worldclim.org/data/bioclim.html (Fick & Hijmans, 2017). We downloaded a raster file of edaphic data for the soil layer between 15 and 30 cm depth using a spatial resolution of 250 m. The 19 bioclimatic variables were downloaded using a spatial resolution of 30 m. Both datasets were cropped to the extent of the sampling area for further analysis. We assessed the correlation between edaphic variables and bioclimatic variables separately through principal component analysis using the function *rasterPCA* of the *RStoolbox* version 0.2.6 (Leutner & Horning, 2019) in order to avoid collinearity between variables. As the edaphic variables showed low correlation (<0.7), we used the original dataset for posterior analysis. Bioclimatic variables showed high correlation; therefore, we based our analyses on the first five PCA components which together explained 95.5% of the variance in the bioclimatic variables (Table S3). We calculated the Mahalanobis distance of the environmental data using the *distance* function of the *ecodist* 2.0.7 package (Goslee & Urban, 2007).

We performed Mantel tests in the R package *ncf* 1.2‐9 (Bjornstad, 2020) using the pairwise *F*_ST_ between populations as genetic distance. We performed simple Mantel tests between genetic and spatial distance, or between genetic and environmental distance, separately for edaphic and bioclimatic variables. Then, we performed partial Mantel tests between genetic and environmental distances in which the spatial distance was used as a controlling factor, and between genetic and spatial distances in which environment was used as a controlling factor.

To further evaluate the association between environmental variables and the genetic divergence among populations, we performed a distance‐based redundancy analysis (dbRDA) (Legendre & Legendre, 2014). This multivariate method combines ordination with multivariate regression approach to find linear relationships between a matrix of predictor variables and a matrix of response variables. The method is relatively robust to collinearity among predictor variables (Wagner & Fortin, 2012) and in the detection of environmental gradients in landscapes (Kierepka & Latch, 2015). In these analyses, we used the environmental dataset as predictors and the scores of the first three axes of a spatial principal components analysis (sPCA) obtained using the *spca* function from *adegenet* package as the genetic variables (Jombart et al., 2008). The sPCA summarizes the genetic variability of a group of individuals while it controls for spatial autocorrelation between them using Moran's I statistic (Jombart et al., 2008). We also used the *pcnm* function of the *vegan* package version 2.5‐7 to calculate spatial predictors for the dbRDA based on the transformation of latitude and longitude of populations (Oksanen et al., 2020). To find the best dbRDA model, we performed a stepwise variable selection based on the Akaike information criterion (AIC) using *ordiR2step* function in *vegan* (Oksanen et al., 2020) and used the variance inflation factor (VIF) to further assess the collinearity of the variables within the selected model. The significance of the models, of the variables in the models, and of the axis of dbRDA was assessed with a permutation test based on 1000 permutations using the *Anova* function in R (R Core Team, 2020). Finally, we estimated the proportion of variance explained by each variable (partial *R* ^2^) using the function *varpart* of the *vegan* package (Oksanen et al., 2020).

## 3 RESULTS

### 3.1 Genetic diversity and structure of *Plathymenia reticulata*

*Plathymenia reticulata* localities showed *A* and *A*_R_ values ranging from 3.9 to 10.0 and from 3.72 to 6.61, respectively, and *H*_O_ and *H*_E_ values ranging from 0.361 to 0.667 and 0.456 to 0.749, respectively (Table 1). Populations of the Cerrado harbored higher diversity than those of the Atlantic Forest for all the diversity indices. In addition, the ecotone localities showed higher diversity than the Atlantic Forest and Cerrado localities; however, the savanna and forest ecotypes in the ecotone exhibited similar overall genetic diversity. The inbreeding coefficients (*F*_IS_) ranged from −0.005 to 0.330 with three localities from the Cerrado, one from the Atlantic Forest, and two in the ecotone showing significant positive *F*_IS_ (*p* < .05 after sequential Bonferroni correction) (Table 1).

**TABLE 1:** Genetic diversity parameters of *Plathymenia reticulata*

Locality	Population code	State	*N*	*A*	*A*_R_	*H*_O_	*H*_E_	*F*_IS_
Cerrado								
Pirenópolis	PRC	GO	28	6.8	5.40	0.562	0.659	**0.149**
Paracatu	PTC	MG	23	6.7	5.40	0.486	0.607	**0.203**
Várzea da Palma	VZC	MG	17	6.0	5.17	0.495	0.602	**0.183**
Overall Cerrado	–	–	68	11.0	6.36	0.519	0.670	**0.226**
Atlantic Forest								
Alto Jequitibá	AJF	MG	22	5.2	4.14	0.361	0.456	**0.214**
Itapebi	IPF	BA	14	3.9	3.72	0.481	0.479	−0.005
Silva Jardim	SJF	RJ	26	5.2	3.96	0.428	0.468	0.087
Overall Atlantic Forest	–	–	62	7.9	4.79	0.416	0.494	**0.159**
Ecotone								
Contagem	COE	MG	12	6.5	6.19	0.630	0.742	0.157
Felício dos Santos	FEE	MG	38	10.0	6.33	0.504	0.749	**0.330**
Ribeirão das Neves	NEE	MG	24	5.4	4.45	0.435	0.567	**0.237**
Parque Estadual do Sumidouro	SUE	MG	29	8.8	6.61	0.667	0.747	0.108
Overall ecotone	‐	‐	103	13.2	7.13	0.549	0.768	**0.286**
Savanna ecotype in ecotone								
Contagem	s‐COE	MG	12	6.5	6.19	0.630	0.742	0.157
Felício dos Santos	s‐FEE	MG	16	7.4	6.07	0.436	0.626	**0.312**
Parque Estadual do Sumidouro	s‐SUE	MG	15	7.6	6.54	0.614	0.660	0.072
Overall ecotone savanna ecotype	–	–	43	11.8	7.09	0.551	0.701	**0.216**
Forest ecotype in ecotone								
Felício dos Santos	f‐FEE	MG	22	6.8	5.17	0.554	0.669	**0.176**
Ribeirão das Neves	f‐NEE	MG	24	5.4	4.45	0.435	0.567	**0.237**
Parque Estadual do Sumidouro	f‐SUE	MG	14	6.5	5.91	0.718	0.769	0.069
Overall ecotone forest ecotype	–	–	60	10.0	6.32	0.549	0.706	**0.224**

Note

*A*, mean number of alleles per locus; *A*_R_, allelic richness; *H*_O_, mean observed heterozygosity; *H*_E_, mean expected heterozygosity; *F*_IS_, inbreeding coefficient. Boldface numbers represent significant *F*_IS_ values (*p* < .05).

AMOVA showed high divergence among localities (*F*_ST_ = 0.187; Table S4). The hierarchical AMOVA showed that most of the variation among localities is explained by the divergence between ecotypes (*F*_CT_ = 0.222 and *F*_ST_ = 0.307). When the hierarchical AMOVA was performed considering localities of the Atlantic Forest and the Cerrado only, the part of total variation attributed to divergence between ecotypes was even higher (*F*_CT_ = 0.337 and *F*_ST_ = 0.396) (Table S4). Pairwise *F*_ST_ values (*p* < .05 for all values) were higher between localities from different biomes (*F*_ST_ = 0.327–0.402) than within biomes (*F*_ST_ = 0.072–0.099) and showed large variation in the ecotone (*F*_ST_ = 0.053–0.316, Table S5).

**FIGURE 2:**
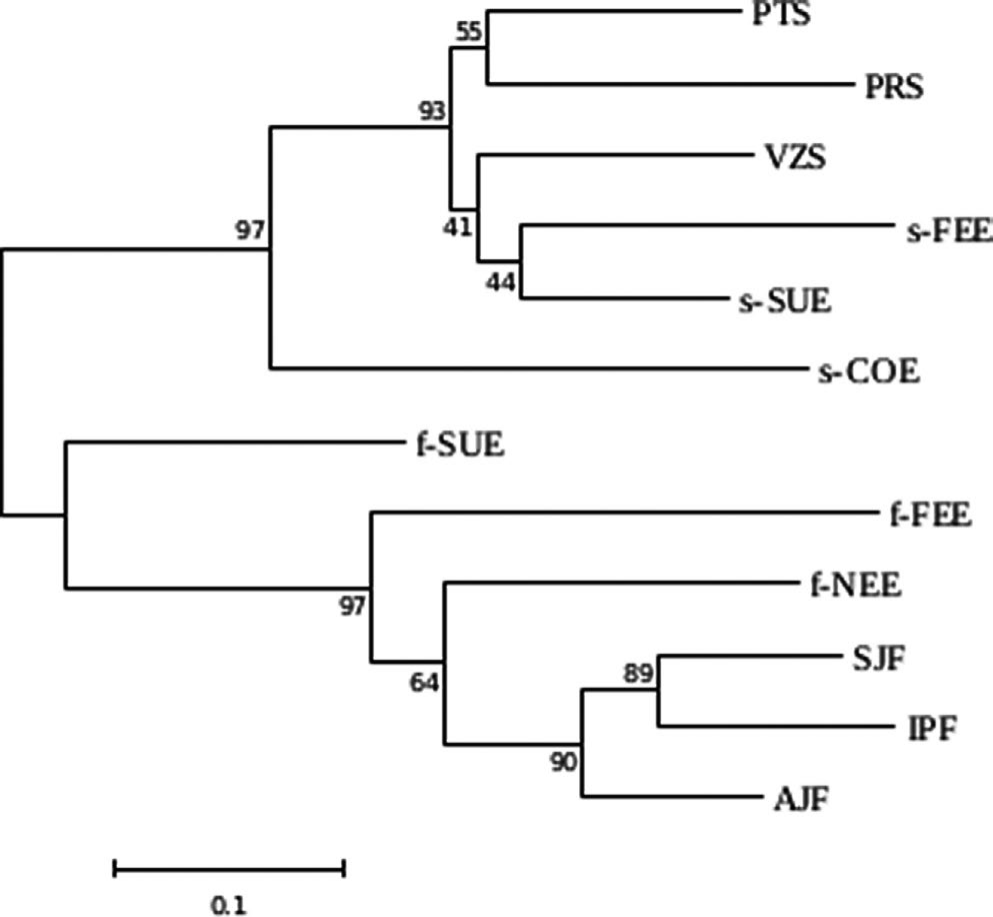
Neighbor‐joining tree based on *D*_ST_ genetic distance among *Plathymenia reticulata* populations with bootstrap support of nodes

The neighbor‐joining (NJ) tree showed that the savanna and forest ecotypes are highly divergent from each other (Figure 2). Both groups showed bootstrap values above 90% indicating a high support for the grouping of these populations. Furthermore, the NJ tree showed that the forest populations of the ecotone were divergent from the populations of the Atlantic Forest (Figure 2).

**FIGURE 3:**
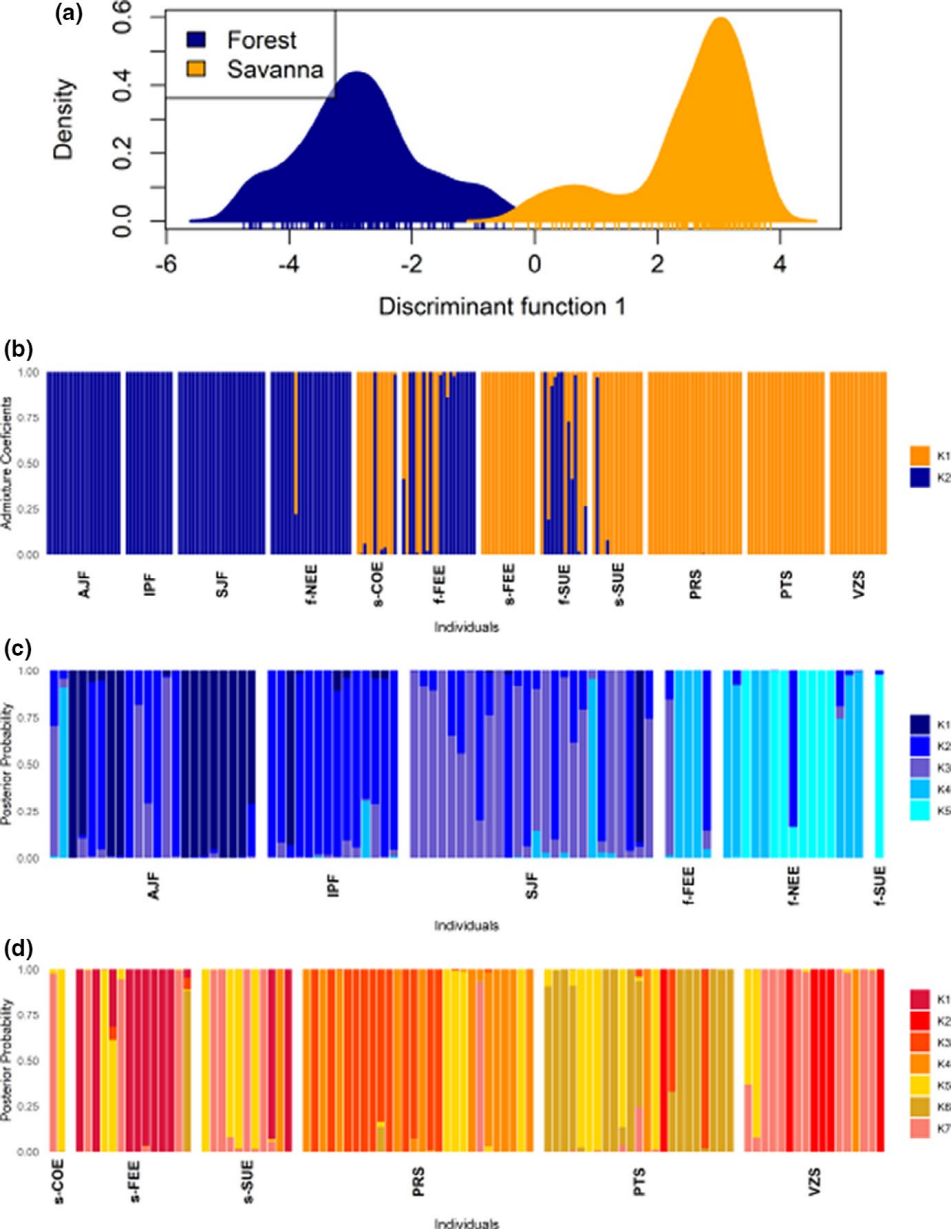
(a) Distribution of the score values of individuals of each ecotype of *Plathymenia reticulata* based *k*‐means grouping and classification with Discriminant Analysis of Principal Components (DAPC). (b) Bar plot showing individual membership probabilities based on DAPC. (c) Bar plot showing individual membership probabilities based on DAPC for pure individuals from the forest ecotype. (d) Bar plot showing individual membership probabilities based on DAPC for pure individuals from the savanna ecotype

**FIGURE 4:**
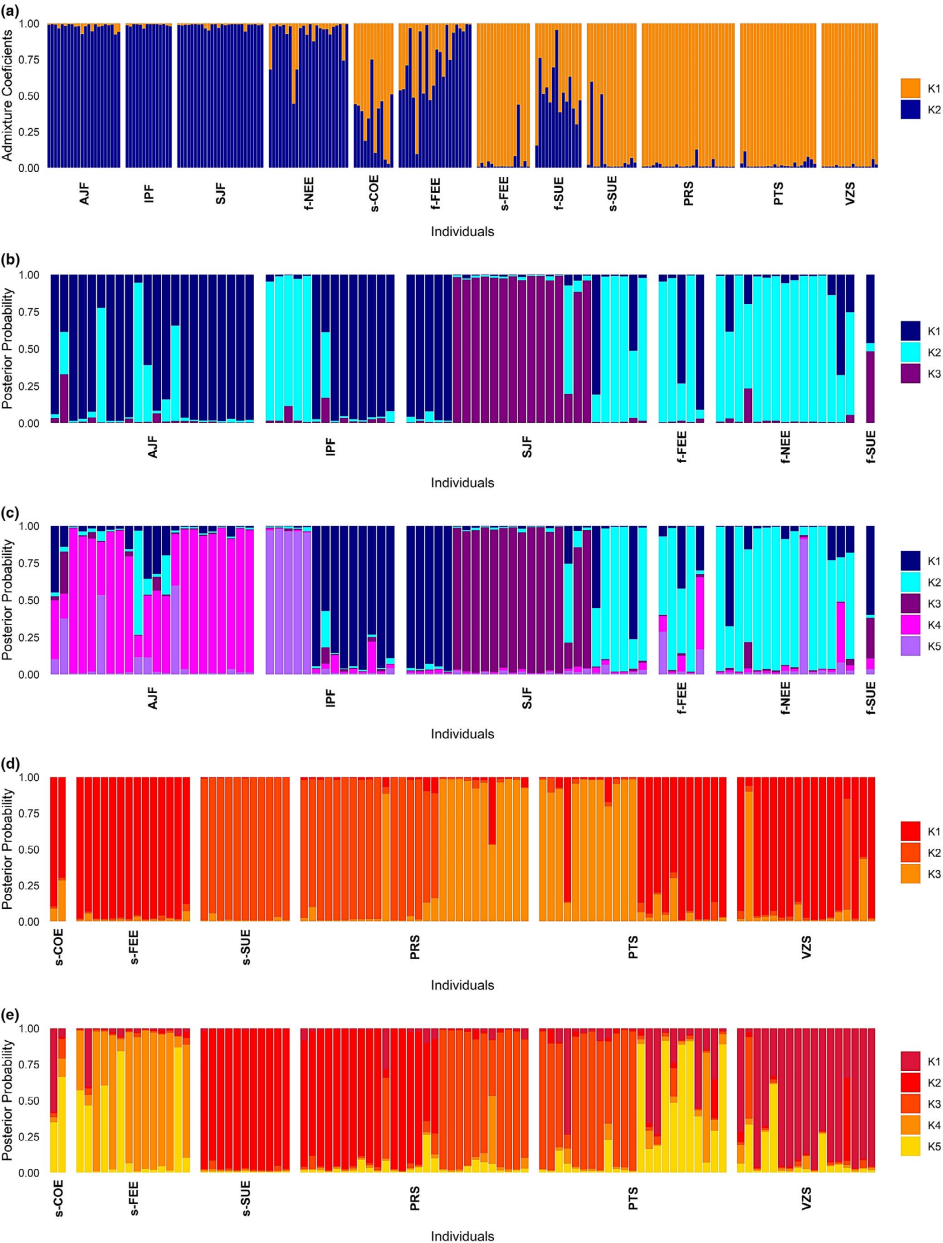
Population structure based on based on the Bayesian clustering method of STRUCTURE. (a) Bar plot showing individual admixture proportions in *K* = 2 genetic groups for all individuals of *Plathymenia reticulata*. Bar plots showing individual admixture proportions for (b) *K* = 3 and (c) *K* = 5 genetic groups for pure individuals of the forest ecotype. Bar plots showing individuals admixture proportions for (d) *K* = 3 and (e) *K* = 5 genetic groups for pure individuals of the savanna ecotype

The number of clusters that best explained the genetic structure in the *P*. *reticulata* dataset was *K* = 2 based on the sequential *k*‐*means* clustering method. The DAPC analysis based on this grouping indicated that most of the genetic divergence in *P*. *reticulata* occurs between forest and savanna ecotypes (Figure 3a). Individuals with intermediate posterior membership probabilities between forest and savanna ecotypes were observed in all ecotone localities, suggesting admixture in the ecotone (Figure 3b). The Bayesian clustering method STRUCTURE showed a similar pattern of genetic divergence in *P*. *reticulata*. The Δ*K* method of Evanno et al. (2005) clearly indicated *K* = 2 as the highest hierarchical level of genetic structure with a separation of *P*. *reticulata* in two genetic groups representing the forest and savanna ecotypes (Figure S2a; Figure 4a). Individuals from localities in the Cerrado and Atlantic Forest did not show admixture, whereas admixture was frequent in individuals from the ecotone (Figure 4a), considering a *Q* value higher than 0.850 (or lower than 0.150) as a threshold for a pure individual. The highest proportion of admixed individuals was found in f‐SUE (86% of individuals) and f‐FEE (41% of individuals) in the forest ecotype and in s‐COE (67% of individuals) in the savanna ecotype.

In agreement with the STRUCTURE and DAPC, the recent migration rates between ecotypes estimated in BayesAss were high in the ecotonal region (Table 2). The migration rate estimates were higher into the forest ecotype in two of three ecotonal population pairs sampled, with values ranging from 0.024 to 0.119 into the forest ecotype and values ranging from 0.030 to 0.061 into the savanna ecotype (Table 2). When considering the whole sample, which included ecotone, Cerrado, and Atlantic Forest localities, the migration rate into the forest ecotype was also higher than that into the savanna ecotype with values of 0.040 (SD = 0.01) and 0.024 (SD = 0.09), respectively, and the same was true for the analysis restricted to ecotone localities (Table 2).

**TABLE 2:** Recent immigration rates (*m*) between forest and savanna ecotypes of *Plathymenia reticulata* estimated by BayeAss

Parameter	Mean	SD
All localities		
*m* to forest	0.040	0.010
*m* to savanna	0.024	0.009
All ecotonal localities		
*m* to forest	0.072	0.019
*m* to savanna	0.057	0.021
COE/NEE localities		
*m* to forest	0.024	0.019
*m* to savanna	0.054	0.035
FEE locality		
*m* to forest	0.039	0.022
*m* to savanna	0.030	0.023
SUE locality		
*m* to forest	0.119	0.076
*m* to savanna	0.061	0.031

In the evaluation of the structure using only the pure individuals of each ecotype in DAPC, we found *K* = 5 in the forest and *K* = 7 in the savanna ecotype. In the forest ecotype, all the localities showed a prevailing genetic cluster despite showing high proportions of shared ancestry mainly among the three populations of the Atlantic Forest and among the three ecotonal localities (Figure 3c). In the savanna ecotype, individual assignment probabilities to the clusters were high, but clusters were shared among localities (Figure 3d). The Δ*K* method of Evanno et al. (2005) on the STRUCTURE analysis of the pure individuals of each ecotype showed *K* = 3 within both forest and savanna ecotypes (Figure 4, Figure S2b,c). Using the criteria of StructureSelector, *K* = 5 was the best number of clusters within both ecotypes (Figure 4, Figure S3a,b). Both ecotypes displayed a genetic structure that was partially congruent with the geographic localities, though most localities featured more than one cluster as well as admixed individuals, especially those of the forest ecotype (Figure 4).

### 3.2 Genotypic composition of *Plathymenia reticulata* in the hybrid zone

**FIGURE 5:**
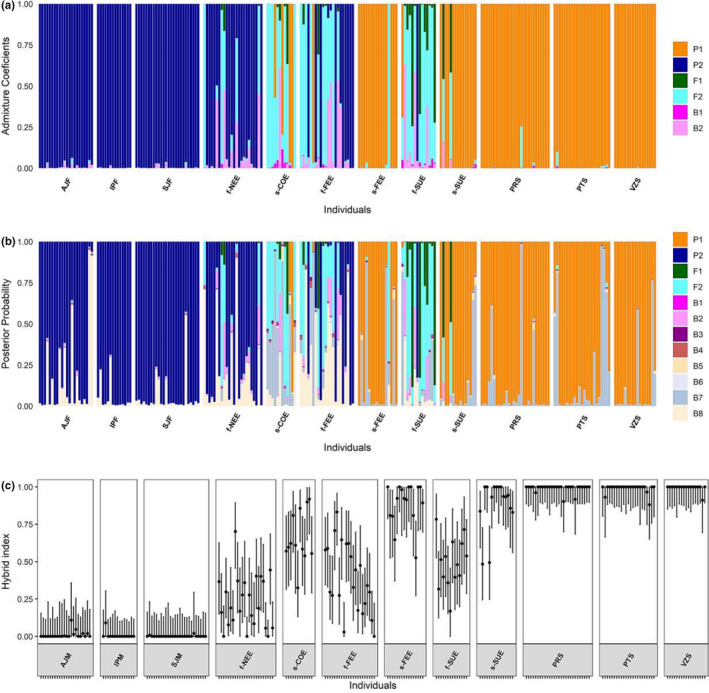
Genotypic composition of *Plathymenia reticulata*. (a) Genotypic assignment of each individual in two parental populations and four hybrid classes based on NewHybrids. (b) Genotypic assignment of each individual in the six mentioned genotypic classes and six advanced generation hybrids based on NewHybrids. (c) Hybrid index estimated for each individual. P1 = savanna ecotype, P2 = forest ecotype, F1 = P1 × P2, F2 = F1 × F1, B1 = P1 × F11, B2 = P2 × F1, B3 = P1 × F2, B4 = P2 × F2, B5 = P1 × (F1 × P1), B6 = P2 × (F1 × P2), B7 = P1 × (P1 × F2), B8 = P2 × (P2 × F2)

The classification of *P*. *reticulata* individuals in the NewHybrids analysis supported, with high posterior probability (PP > 0.90), a pure parental composition for most individuals in the areas of the Cerrado and Atlantic Forest (Figure 5a). In the ecotone, 18 individuals were classified as F2, with 12 of them resembling the forest ecotype and 6 the savanna ecotype (Figure 5a). Twenty additional individuals were identified as hybrids with posterior probability values between 0.4 and 0.8 for different hybrid genotypic classes involving F1, F2, and backcross to the forest ecotype (B2), but the posterior probability of backcross to the savanna ecotype (B1) never exceeding 10% (Figure 5a). The localities with the highest proportions of F2 individuals were s‐COE, f‐FEE, and f‐SUE with 41%, 27%, and 21% of F2 individuals, respectively (Figure 5a). The classification in 12 genotypic classes assigned as pure, a proportion of 0.636–0.857 of individuals from Atlantic Forest and Cerrado localities, respectively (Figure 5b). Three individuals showed high posterior probabilities of being classified as second‐generation backcrosses (B7 or B8) in AJF, PRS, and PTS localities (Figure 5b). Furthermore, in f‐SUE, 14% of individuals were classified as F1 when using 12 genotypic classes. The high proportion of individuals classified as later generation hybrids in this analysis may indicate that the analysis using six genotypic classes misclassified later generation hybrids as pure.

**FIGURE 6:**
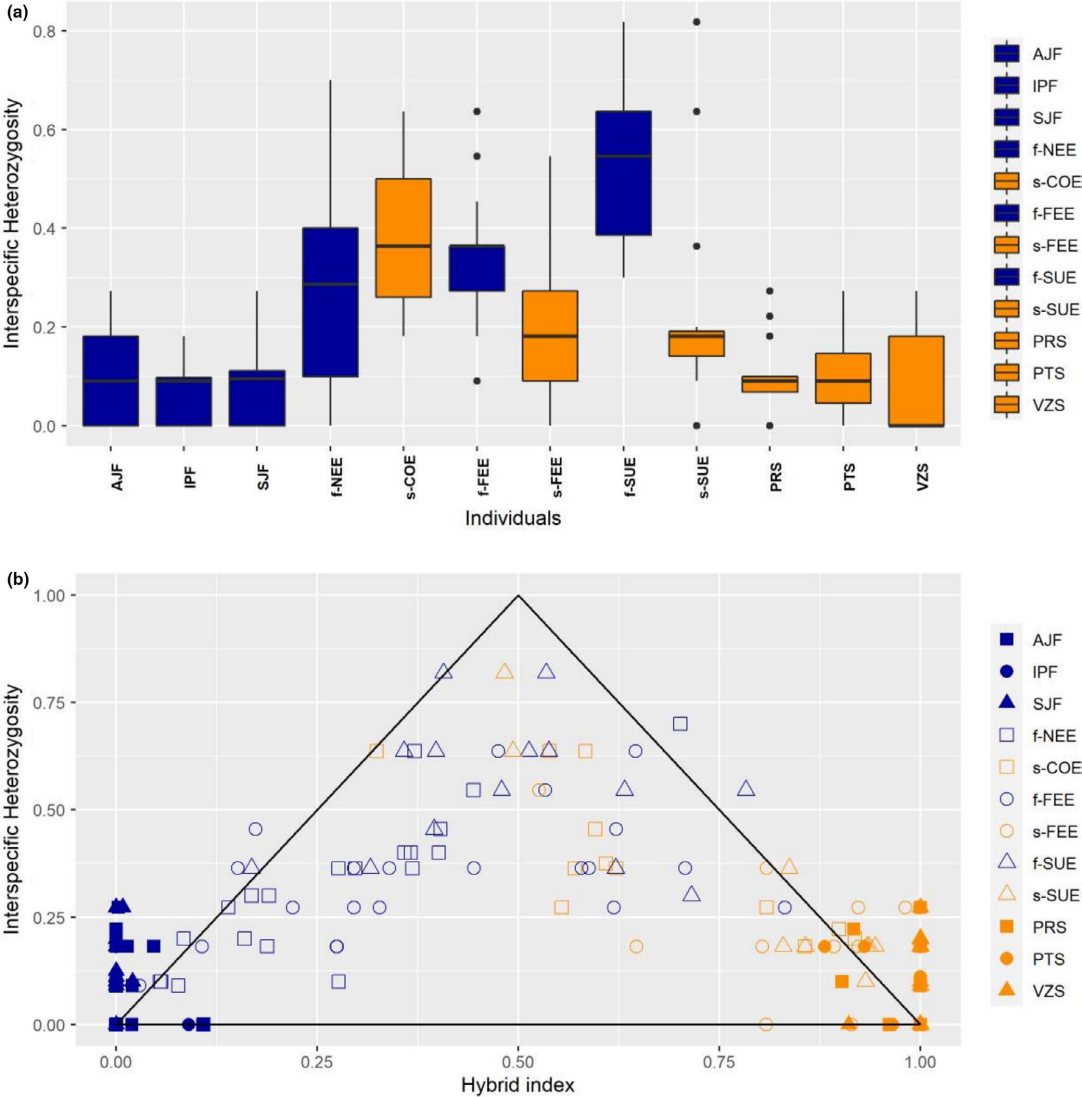
(a) Boxplot showing the distribution of interecotype heterozygosity values by sampling localities of *Plathymenia reticulata*. (b) Hybrid index × interecotype heterozygosity values showing the composition of hybrids in the hybrid zone

Consistent with the NewHybrids results, the mean hybrid index (Figure 5c) of the ecotone localities indicated high admixture levels with mean values per locality ranging from 0.083 to 0.771 and lower bounds of the 95% confidence (CI) interval ranging from 0.024 to 0.540 and upper bounds of the 95% CI ranging from 0.279 to 0.914. The proportion of individuals per population with a hybrid index between 0.200 and 0.800 ranged from 0.133 to 0.929 in the ecotone localities, with the highest values in COE, NEE, and f‐SUE populations (Figure 5c). In comparison, the localities of both biomes showed mean hybrid index values ranging from 0.990 to 0.999 in the Atlantic Forest and from 0.008 to 0.010 in the Cerrado (Figure 5c). The individual interecotypic heterozygosity (*Q*_12_) ranged from 0.00 to 0.818, with the ecotonal localities showing higher mean values (*Q*_12_ ranging from 0.261 to 0.403) than the localities of each biome (*Q*_12_ ranging from 0.071 to 0.104) (Figure 6a). In addition, the interecotypic heterozygosity × hybrid index plots showed a very dispersed distribution for individuals, which indicates that the ecotonal localities mostly comprise later generation hybrids and backcrossed individuals (Figure 6b). A population comprising mostly F1s would show a mean hybrid index closer to 0.5 with interecotypic heterozygosity values closer to the maximum value of 1.0. Finally, some individuals showed higher values than expected for interecotypic heterozygosity with values above the triangle bounds. This occurred mainly in the individuals of localities from Cerrado and Atlantic Forest suggesting that homoplasy may be found in the microsatellite dataset.

### 3.3 Evaluation of hybrid assignment in NewHybrids

The model evaluation using NewHybrids with six simulated genotypic classes showed an average proportion of correct assignments of pure parental individuals of almost 91% when setting a threshold of 0.85 (Table S6; Figure S4). Only the F2 category showed a proportion of correct assignments lower than 50%, while F1, B1, and B2 displayed 79%, 66%, and 65% correct assignments, respectively. When using 14 simulated crosses for the assignment in 12 genotypic classes, a very low proportion of correct assignment of pure individuals was found, with most of them showing values of posterior probability of assignment below 0.85 (Table S6; Figure S4). The classification was better for the hybrid classes, especially for F1 and F2 with 57% and 59% of individuals correctly assigned to their respective categories (Table S6). The classification of backcrosses never exceeded 10% of correct assignments suggesting that our data could not resolve later generation hybrids correctly, especially for second‐generation backcrosses (Table S6). These results indicate a relatively high capacity in the differentiation between admixed and nonadmixed individuals, but a poorer capacity to differentiate among advanced generation hybrids (F2 and backcrosses). Finally, these simulations let us assume that some individuals classified as F2 in the NewHybrids analysis with six genotypic classes could in fact be backcrosses (Figure S5). The evaluation of the performance of STRUCTURE and *introgress* to identify and classify hybrids based on simulated data is presented in Appendix S1.

### 3.4 Drivers of divergence of *Plathymenia reticulata* ecotypes

Simple Mantel tests indicated that the dataset displayed both a signature of isolation by distance and of isolation by environment (Table 3). Genetic distance showed a higher correlation with bioclimatic distance (*r* = .381, *p* = .006) than with spatial distance (*r* = .307, *p* = .009) or soil distance (*r* = .308, *p* = .023). The partial Mantel test showed that the correlation of the bioclimatic distance with the genetic distance remained significant when controlling for geographical distance (*r* = .260, *p* = .035, Table 3). Other partial Mantel tests were not significant.

**TABLE 3:** Mantel tests showing the correlation between genetic distance and environmental distances (soil and climate) between populations of *Plathymenia reticulata*

Soil	Gen × Soil	Gen × Geo	Gen × Soil (geo)	Gen × Geo (soil)
Correlation	0.308	0.307	0.163	0.163
*p*	.023	.009	.137	.104
Climate	Gen × Clim	Gen × Geo	Gen × Clim (geo)	Gen × Geo (clim)
Correlation	0.381	0.307	0.260	0.111
*p*	.006	.008	.035	.196

Note

Control variables are given within brackets for partial Mantel tests.

**FIGURE 7:**
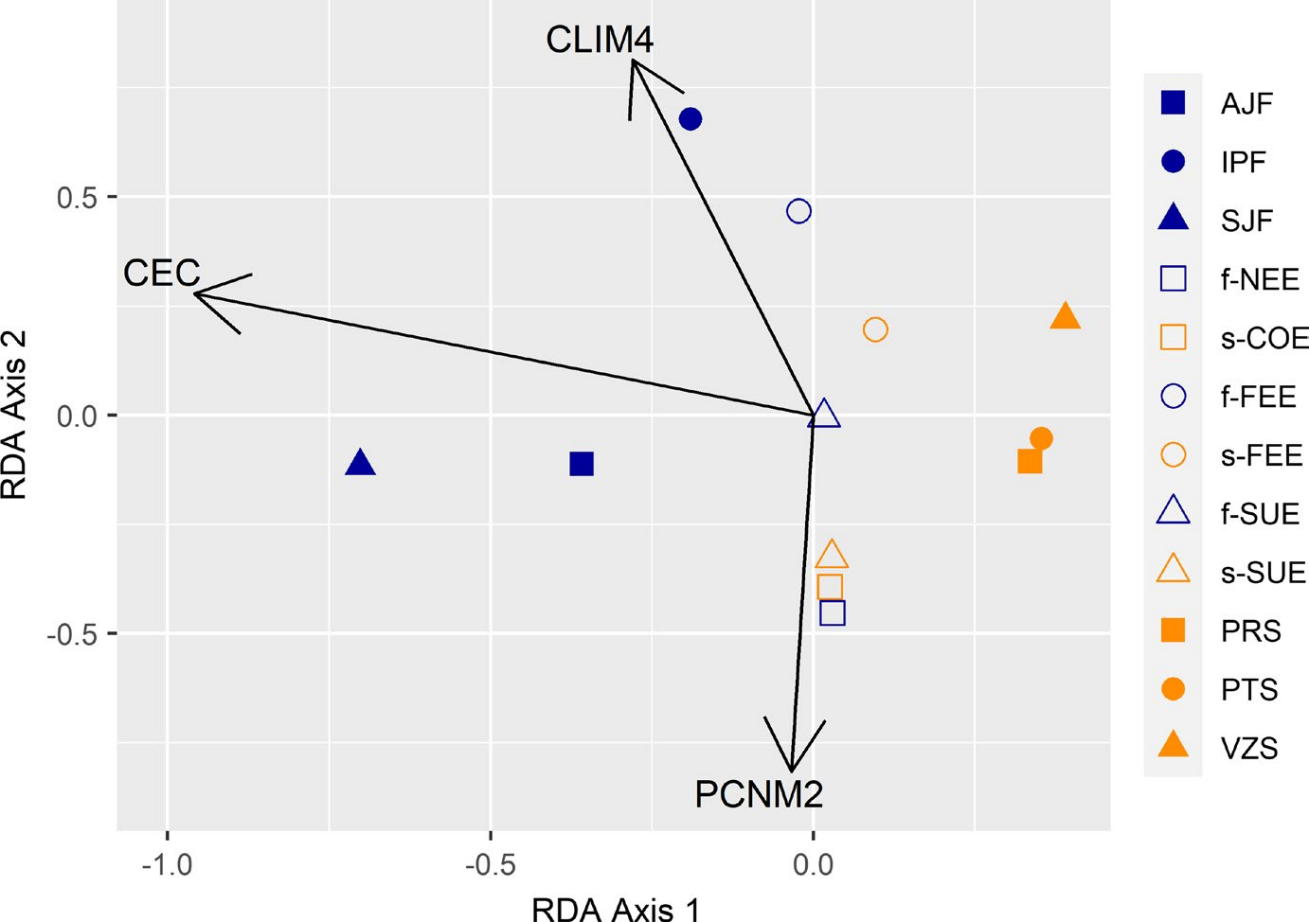
Scatter plot showing the distribution of redundancy analysis (RDA) scores of *Plathymenia reticulata* populations and the correlation of environmental variables with the RDA axis

The sPCA test indicated a significant global structure (*p* = .042) with the first three axes showing significant divergence between the *P*. *reticulata* populations (Figure S5). The dbRDA analysis identified an association of genetic divergence based on sPCA with Cation Exchange Capacity (CEC), the fourth synthetic variable of the climatic PCA (CLIM4), and with spatial distance between populations (PCNM2) (Table 4). CLIM4 was mainly associated with Precipitation of the Warmest Quarter (bio18, loading = −0.645), Mean Diurnal Range Temperature (bio2, loading = 0.398), and Precipitation of the Wettest Quarter (bio16, loading = 0.309) (Table S3). The CEC and PCNM2 were associated with the first dbRDA axis with loadings of −0.958 and −0.815, respectively. The second dbRDA axis was associated with the CLIM4 variable with a loading value of 0.813 (Table S8). The estimated partial adjusted *R* ^2^ showed that CEC was the variable with the highest explanatory power, with CLIM4 being the second (Table 4). The first axis of dbRDA separated the populations of the Cerrado from those of the Atlantic Forest with the ecotonal populations being intermediary (Figure 7).

**TABLE 4:** Results of model selection and redundancy analysis testing the association between allelic variation of *Plathymenia reticulata*, spatial distance, and environmental variables

Variable	df	Sum of Sqs	*F*	Pr(>*F*)	Ajusted.*R* ^2^	VIF
CEC	1	10.225	7.814	0.0001	0.301	2.228
CLIM4	1	6.793	5.191	0.0007	0.227	1.368
PCNM2	1	5.514	4.214	0.0029	0.102	2.326
Residual	8	10.469				

Abbreviations: CEC, Cation Exchange Capacity; CLIM4, Climatic Axis obtained from PCA; PCNM2, Spatial axis obtained from Principal Coordinates Neighboring Matrix; VIF, Variance Inflation Factor.

## 4 DISCUSSION

Our study reveals genetic divergence between savanna and forest ecotypes of *P*. *reticulata* and gene flow between ecotypes across ecotonal areas between the Brazilian Cerrado and Atlantic Forest, characterizing a hybrid zone. Hybrid individuals mostly corresponded to second‐generation hybrids or backcrosses toward the forest ecotype. We observed asymmetric gene flow between ecotypes, which was higher into the forest ecotype. Our data also suggest that besides the geographical distance, climatic and edaphic variables partially explain the genetic divergence between the ecotypes. The ecotonal areas harbor high genetic diversity relative to the Atlantic Forest and Cerrado biomes for *P*. *reticulata* and can be an important source of variation for the species, mainly for the forest ecotype.

### 4.1 Genetic divergence between savanna and forest ecotypes of *Plathymenia reticulata*

The analyses of the genetic structure and divergence of *P*. *reticulata* showed a clear genetic distinction between savanna and forest ecotypes, consistent with our a priori classification of individuals based on morphological characters of the tree trunk, bark, height, and environment of the trees. The genetic distinction between ecotypes was reinforced by the fact that even in the ecotonal localities this division was recovered using NJ trees, DAPC, and STRUCTURE analyses. Populations from the forest ecotype in the ecotone are more genetically related to populations from the Atlantic Forest, whereas populations of the savanna ecotype in the ecotone are more related to populations from the Cerrado, despite high genetic admixture in the ecotone. These genetic data are consistent with ecological studies that showed that the two ecotypes maintain their morphophysiological traits in the ecotonal areas, though ecotonal individuals are less dissimilar to the alternative ecotype than when comparing distinct ecotypes in their specific habitats (Goulart et al., 2005, 2006, 2011; Toledo et al., 2012). Differences between ecotypes in several of these traits were demonstrated to be genetically determined when progenies were cultivated in a common garden experiment with different light conditions (Goulart et al., 2011).

We found an association of edaphic and climatic variables with *P*. *reticulata* genetic divergence that was in some instances stronger than the divergence explained by spatial distance between localities, suggesting a pattern of isolation by environment (IBE). The genetic divergence was associated with Cation Exchange Capacity (CEC) of the soil and with Precipitation of the Warmest Quarter, Precipitation of the Wettest Quarter, and Mean Diurnal Range Temperature (summarized in the synthetic variable CLIM4). We detected IBE using SSR markers that are considered neutral, congruent with a scenario of genome‐wide hitchhiking following long‐lasting selection that may have affected a small fraction of the genome, in combination with assortative mating (Andrew et al., 2012; Sexton et al., 2014, 2016). The high proportion of variance explained by CCE suggests that the differences in soil between savanna and forest can be important in the divergence in *P*. *reticulata*. Differences in soil can affect plant growth strategy and be an important driver of plant functional traits and adaptive divergence even at small spatial scales (Schmitt et al., 2021), as is the case in our study where patches of savanna and forest form a mosaic of abrupt habitat shifts within meters in ecotonal areas (Lambers et al., 2011; Reich et al., 2003). Previous studies have shown the adaptive significance of intraspecific variation for population persistence in forest–savanna ecotones, where different resource‐use strategies are required in each habitat (Maracahipes et al., 2018; Silva et al., 2019). Soils of Atlantic Forest localities are more fertile and have higher CEC than the Cerrado localities, with ecotonal localities showing intermediary values. CEC can be used as a proxy of soil fertility and nutrient retention, as it relates to the soil's capacity to provide calcium, magnesium, and potassium for plants (https://www.fao.org/; https://www.soilquality.org.au/). Therefore, the high genetic variation we observed across very close‐by sites in the ecotone could suggest high adaptive potential, assortative mating of adapted individuals to local soil, and selection against maladapted individuals into the alternative habitat, leading to a pattern of IBE.

Regarding bioclimatic variables, the Mean Diurnal Range in temperature, the Precipitation of the Wettest Quarter, and the Precipitation of Warmest Quarter were associated with genetic divergence and are related with extremes of temperature within a day and the seasonality in rainfall along the seasons, respectively. Associations between genetic divergence and the Mean Diurnal Range (Buzatti et al., 2019) or between genetic diversity and Precipitation of the Wettest Quarter (Ribeiro et al., 2016) were already found for *Qualea grandiflora* and *Annona coriacea*, respectively, two of the most common tree species of the Cerrado. A similar association was also found in an annual herb, *Brasilianthus carajensis*, in a mountain savanna in *Canga* plateaus within the Amazon Forest (Silva et al., 2020). The Cerrado is a more stressful environment than the Atlantic Forest in many ways, as it is, for example, characterized by a stronger and more prolonged drought season. Accordingly, in *P*. *reticulata*, previous studies on some of the sampling locations of the present study (COE and NEE in Goulart et al., 2006, and SUE in Toledo et al., 2012) showed that savanna and forest ecotypes are differentiated for drought response traits even in ecotonal areas. Differences between savanna and forest ecotypes concern also the vegetative phenology, with leaf shed beginning earlier in the Cerrado than in ecotonal areas, and later in the forest (Goulart et al., 2005). In addition, the forest ecotype shows higher stem radial increment, positively related to increasing precipitation, relative to the savanna ecotype, which shows lower midday water potential during the dry season relative to the forest ecotype (Toledo et al., 2012). Trait divergence thus appears in accordance with environmental variation and genetic divergence in *P*. *reticulata*. Conclusive evidence on the genomic signature of natural selection in the species could be obtained from genome‐wide data that might reveal contrasted differentiation of neutral versus potentially adaptive alleles in ecotype divergence.

The high genetic divergence between ecotypes accounting for 22.2–33.7% of the total genetic variance (Table S2) raises questions about the taxonomy of the genus. In comparison, divergence between two *Hymenaea* (Fabaceae) tree species from the Atlantic Forest and Cerrado accounted for 26% of the total genetic variance based on seven nuclear microsatellites (mean *H*_E_ = 0.600, Resende‐Moreira et al., 2017). In *Eucalyptus globulus*, conversely, differentiation between ecotypes was lower, with pairwise *F*_ST_ values ranging from 0.03 to 0.21 based on using 12 nuclear microsatellites (mean *H*_E_ = 0.84, Foster et al., 2007). Pairwise *F*_ST_ values between *P*. *reticulata* localities of the Atlantic Forest and the Cerrado ranged from 0.327 to 0.402. This relatively high level of genetic divergence between *P*. *reticulata* ecotypes associated to morphophysiological differences found in several previous studies suggests that they could represent incipient species. These observations are in accordance with the older taxonomic status that considered the *Plathymenia* genus as represented by two species, *P*. *reticulata* Benth in the Cerrado and *P*. *foliolosa* Benth in the Atlantic Forest (Heringer, 1956).

### 4.2 Characterization of *Plathymenia reticulata* in the hybrid zone

The high levels of genetic admixture in several ecotonal areas, with a mean of 40% of hybrids per locality, characterize these areas as a hybrid zone. Most hybrid individuals were classified as later generation hybrids with none showing high probabilities to be F1s and very few showing posterior probability between 0.5 and 0.9 to be classified as F1. Classifying hybrids in genotypic classes based on a few microsatellite markers is challenging, as shown by our simulation study which illustrated the difficulty of distinguishing later generation hybrids from pure individuals depending on the chosen assignment threshold. It should be noted that some studies have shown that individuals previously classified as F2 based on microsatellite data were in fact classified as F1s when using genomic data (Gramlich et al., 2018; Lindtke et al., 2014). In the microsatellite dataset, these misclassified individuals showed levels of interspecies ancestry lower than those expected for F1 hybrids, but unusually higher than those predicted for later generation hybrids. In our dataset, some individuals showed this unusual pattern of high interecotype heterozygosity, which may suggest that our analysis underestimated the number of F1s. Therefore, a study based on genomic data may better estimate the number of early generation hybrids along the *P*. *reticulata* hybrid zone. Such a study will also increase the power of classifying later generation hybrids and help shed light on which hybridization events and backcross scenarios provide opportunities for introgression between the ecotypes.

We observed higher gene flow in direction of the forest ecotype than toward the savanna ecotype. Several causes such as demographic processes related to range expansion may cause asymmetric hybridization (Currat et al., 2008; Field et al., 2010). The savanna ecotype populations could be expanding into forested habitats and the interbreeding between the savanna and forest ecotypes in these areas would lead to the invasion of genes of forest populations by genes of the savanna ecotype. Differences in dispersal capacity may also cause differential introgression with the populations with higher dispersal capacity promoting the spread of alleles to the other populations (Yamamoto et al., 2019). The *P*. *reticulata* forest ecotype showed seed structures associated with higher dispersal capacity (Goulart et al., 2006), which can indicate that this factor is not the better explanation for the differential introgression. However, the occurrence of the savanna ecotype in open habitats may provide opportunities for dispersal across longer distances which may lead to the capacity of savanna ecotypes to colonize surrounding forested areas. Natural selection can also act as a driver of differential introgression (Lexer et al., 2005; Whitney et al., 2006). Ecological barriers, that is, a harsh environment, may effectively reduce the gene flow into savanna populations. Another hypothesis to explain the differences in gene flow could be related to the longer juvenile stage of forest individuals compared to the savanna individuals in which frequent fires appear to select for earlier reproductive maturity (Hoffmann & Franco, 2003). Furthermore, the Cerrado biome besides typical savanna vegetation presents more dense wood formations (Cerradão) and riparian forests where *P*. *reticulata* savanna individuals can be found with more similar characteristics to forest individuals. Thus, the higher effective gene flow toward the forest ecotype may be due to the fact that some Cerrado individuals are more able to colonize and survive in forest habitat.

Novaes et al. (2010) suggested that *P*. *reticulata* lineages diversified from its center of distribution in the Cerrado shaped by climatic changes in the Pleistocene. The lower genetic diversity of the forest ecotype in the Atlantic Forest, compared to the savanna ecotype, suggests recent expansion of *P*. *reticulata* toward the Atlantic Forest possibly involving founder effects that led to the loss of genetic diversity. The spatial dynamics of Atlantic Forest and Cerrado biomes during glacial cycles in the Pleistocene, with the Cerrado progressing eastward into the forests during drier and colder periods (Behling, 1998; Behling & Negrelle, 2001; Meneses et al., 2013), may have provided opportunities for the expansion of *P*. *reticulata* from the savanna toward previously forested areas. Phylogeographical studies suggest similar dynamics of range expansions and contractions in tree species of the Cerrado and Atlantic Forest during the Pleistocene (Buzatti et al., 2017; Buzatti et al., 2019; Leal et al., 2016; Novaes et al., 2013; Ribeiro et al., 2011).

Gene flow in vicariant species such as *P*. *reticulata* with subspecific taxa typical from forests and savannas was also identified in a few other tree species in savanna–forest boundaries including ecotonal and riparian forests in the Cerrado biome (Cavallari et al., 2010; Muniz et al., 2020; Resende‐Moreira et al., 2017). This can suggest that hybridization in the ecotonal region between Atlantic Forest and Cerrado can be a relevant evolutionary process increasing the genetic diversity of the species of these biomes. The study of other closely related species of other groups can show how common and phylogenetically widespread is the gene flow between lineages of Cerrado and Atlantic Forest.

### 4.3 Implications for conservation

The highest genetic diversity found here in *Plathymenia* populations of the savanna–forest ecotone may be due to the hybridization between ecotypes/species across this area. This is a pattern commonly found in hybrid zones where the admixture between lineages increases genetic diversity (Carlson et al., 2017; Eaton et al., 2015; Y. Li et al., 2016; Zalapa et al., 2010). Therefore, these ecotonal areas are an important source of genetic diversity for *Plathymenia*, mainly for Atlantic Forest populations that show low genetic diversity. The Atlantic Forest has a history of extensive habitat loss and fragmentation and currently remains mostly in small and isolated patches (Ribeiro et al., 2009). In comparison, the deforestation in the Cerrado, though very quick, was only significant from the middle of the twentieth century (Silva et al., 2006). The low genetic diversity of the Atlantic Forest populations indicates that they may have lower adaptive potential and could be severely impacted by ongoing habitat loss and fragmentation in the Atlantic Forest. The forest ecotype, previously considered as *P*. *foliolosa*, is considered vulnerable in the IUCN red list (World Conservation Monitoring Centre, 1998). The conservation efforts for the forest ecotype should be increased and this taxon should be considered a different management unit from the savanna ecotype. Thus, in addition to the conservation efforts in the Atlantic Forest and Cerrado areas, our data point to the importance of including ecotonal areas between these biomes, which could carry alleles to augment the genetic diversity in the Atlantic Forest.

## 5 CONCLUSION

Based on multiple independent approaches, with distinct assumptions, we showed that the ecotonal areas between the Cerrado and the Atlantic Forest constitute a hybrid zone between the genetically differentiated forest and savanna ecotypes of *P*. *reticulata*. Our results suggest the suitability of the ecotonal areas for studies of evolutionary divergence and speciation. The two *P*. *reticulata* ecotypes should be considered as different species or at least two different management units with special attention to the forest ecotype. The dynamics of ecotonal areas, as evidenced here, can have a significant role in the origin of the high biodiversity in the Cerrado and Atlantic Forest, highlighting the importance of these areas as hotspot of genetic diversity and conservation.

## CONFLICT OF INTEREST

The authors declare no conflict of interest.

## AUTHOR CONTRIBUTIONS

**André Carneiro Muniz:** Formal analysis (lead); investigation (equal); methodology (equal); writing – original draft (lead); writing – review and editing (equal). **Ricardo José Gonzaga Pimenta:** Formal analysis (supporting); methodology (equal). **Mariana Vargas Cruz:** Formal analysis (supporting); investigation (equal); methodology (equal). **Jacqueline Gomes Rodrigues:** Investigation (supporting); methodology (equal). **Renata Santiago de Oliveira Buzatti:** Investigation (supporting); methodology (equal). **Myriam Heuertz:** Formal analysis (lead); investigation (equal); methodology (equal); writing – review and editing (equal). **Jose P. Lemos‐Filho:** Conceptualization (equal); funding acquisition (equal); investigation (equal); resources (equal); writing – review and editing (equal). **Maria Bernadete Lovato:** Conceptualization (equal); formal analysis (equal); funding acquisition (lead); investigation (lead); methodology (equal); project administration (lead); supervision (lead); writing – review and editing (equal).

## Supporting information

Supplementary Material

## Data Availability

Genotype data, geographical coordinates, and scripts to simulate individuals in distinct genotypic classes will be archived in Dryad under https://doi.org/10.5061/dryad.905qfttn0.
