# Supplementary material for: Hybrid zone of a tree in a Cerrado/Atlantic Forest ecotone as a hotspot of genetic diversity and conservation
Source: Ecol Evol. 2022 Jan 22;12(1):e8540. doi: 10.1002/ece3.8540 (PMC8803295; doi:10.1002/ece3.8540)
Supplement: Supplementary file 1 — Supplementary Material [file ECE3-12-e8540-s001.docx]

**Original Articles (Primary Research Papers)**

**TITLE:** Hybrid zone of a tree in a Cerrado/Atlantic Forest ecotone as a hotspot of genetic diversity and conservation

**Running title**: Hybrid zone in savanna-forest ecotone

André Carneiro Muniz^1^, Ricardo José Gonzaga Pimenta^1^, Mariana Vargas Cruz^1^, Jacqueline Gomes Rodrigues¹, Renata Santiago de Oliveira Buzatti^1^, Myriam Heuertz^2^, José P. Lemos-Filho^3^ and Maria Bernadete Lovato^1,*^

^1^Departamento de Genética, Ecologia e Evolução, Universidade Federal de Minas Gerais, CP 486, Belo Horizonte, MG 31270-901, Brazil

^2^Biogeco, INRAE, Univ. Bordeaux, 69 route d’Arcachon, 33610 Cestas, France

^3^Departamento de Botânica, Universidade Federal de Minas Gerais, Belo Horizonte, MG 31270-901, Brazil

*Corresponding author

E-mail address: lovatomb@icb.ufmg.br; telephone number: + 55 (31) 34092571; postal address: ^1^Departamento de Genética, Ecologia e Evolução, Universidade Federal de Minas Gerais, CP 486, Belo Horizonte, MG, 31270-901, Brazil

Supplementary table 1: Null allele frequencies for microsatellite markers in each ecotype of each locality of *Plathymenia reticulata* estimated by the Brookfield 1 method in Micro-Checker. Significant P values (P < 0.05) are in bold.

| POP | PRE05 | PRE10 | PRE11 | PRE14 | PRE15 | PRE16 | PR18 | PRE23 | PR30 | PR71 | PR80 |
| --- | --- | --- | --- | --- | --- | --- | --- | --- | --- | --- | --- |
| AJF | 0.039 | **0.205** | **0.080** | 0.030 | 0.073 | 0.051 | 0.051 | 0.045 | -0.001 | -0.006 | 0.052 |
| s-COE | 0.040 | **0.125** | -0.023 | **0.153** | -0.024 | -0.004 | 0.092 | **0.159** | -0.141 | -0.004 | 0.145 |
| s-FEE | **0.129** | 0.023 | **0.157** | -0.040 | **0.229** | 0.093 | 0.031 | **0.270** | 0.092 | **0.111** | -0.002 |
| f-FEE | -0.035 | **0.208** | **0.178** | 0.024 | **0.120** | 0.054 | -0.031 | 0.099 | 0.112 | 0.010 | -0.082 |
| IPF | -0.050 | 0.013 | 0.000 | -0.107 | -0.085 | 0.019 | -0.036 | -0.019 | 0.117 | 0.006 | 0.030 |
| f-NEE | 0.186 | 0.055 | 0.079 | 0.022 | **0.334** | -0.044 | -0.050 | **0.225** | -0.065 | 0.039 | -0.017 |
| PRS | -0.017 | **0.133** | 0.031 | **0.218** | **0.197** | -0.089 | -0.004 | 0.038 | -0.001 | 0.003 | 0.028 |
| PTS | -0.017 | **0.133** | 0.031 | **0.218** | **0.197** | -0.089 | -0.004 | 0.038 | -0.001 | 0.003 | 0.028 |
| SJF | 0.038 | 0.008 | **0.071** | -0.029 | **0.114** | 0.050 | -0.088 | 0.055 | 0.000 | 0.021 | -0.006 |
| s-SUE | 0.068 | 0.086 | -0.047 | 0.024 | 0.008 | -0.016 | -0.053 | 0.091 | -0.010 | 0.015 | -0.008 |
| s-SUE | **0.153** | 0.022 | -0.019 | 0.033 | **0.175** | -0.063 | -0.023 | **0.160** | -0.247 | -0.054 | -0.044 |
| VZS | 0.066 | 0.036 | -0.036 | **0.330** | -0.043 | 0.021 | -0.003 | -0.025 | **0.127** | **0.161** | 0.001 |

Supplementary table 2: Tested genotypic categories in NewHybrids analyses based on Chhatre et al., (2018) showing the expected ancestry proportions of each cross type.

|  |  | |  | | | | |
| --- | --- | --- | --- | --- | --- | --- | --- |
| Genotypic class | Crossing | Expected Ancestry Proportions  AA Aa aA aa | | | | |  |
| Pure | savanna ecotype | 1.000 | | 0.000 | 0.000 | 0.000 |  |
| Pure | forest ecotype | 0.000 | | 0.000 | 0.000 | 1.000 |  |
| F1 | savanna × forest | 0.000 | | 0.500 | 0.500 | 0.000 |  |
| F2 | F1 × F1 | 0.250 | | 0.250 | 0.250 | 0.250 |  |
| F1 Backcross 1 (B1) | F1 × savanna | 0.500 | | 0.250 | 0.250 | 0.000 |  |
| F1 Backcross 2 (B2) | F1 x forest | 0.000 | | 0.250 | 0.250 | 0.500 |  |
| F2 Backcross 1 (B3) | F2 x savanna | 0.500 | | 0.125 | 0.125 | 0.250 |  |
| F2 Backcross 2 (B4) | F2 x forest | 0.250 | | 0.125 | 0.125 | 0.500 |  |
| 1 Backcross × F1 Backcross 1 (B5) | savanna x (F1 x savanna) | 0.750 | | 0.125 | 0.125 | 0.000 |  |
| 2 Backcross × F1 Backcross 2 (B6) | forest x (F1 x forest) | 0.000 | | 0.125 | 0.125 | 0.750 |  |
| 1 Backcross × F2 Backcross 1 (B7) | savanna x (F2 x savanna) | 0.625 | | 0.125 | 0.125 | 0.125 |  |
| 2 Backcross × F2 Backcross 2 (B8) | forest x (F2 x forest) | 0.125 | | 0.125 | 0.125 | 0.625 |  |

Supplementary table 3: Proportion of variance explained by each axis of PCA and loadings of PCA in the climatic dataset indicating the correlation between climatic axis and bioclimatic variables.

| Climatic variables | | | | | |
| --- | --- | --- | --- | --- | --- |
| Proportion of variance explained | | | | | |
|  | Comp.1 | Comp.2 | Comp.3 | Comp.4 | Comp.5 |
|  | 0.576 | 0.160 | 0.117 | 0.0585 | 0.0393 |
|  | Comp.1 | Comp.2 | Comp.3 | Comp.4 | Comp.5 |
| bio1 | 0.268 | 0.252 | 0.079 | 0.004 | 0.078 |
| bio2 | -0.232 | 0.115 | -0.01 | -0.398 | -0.062 |
| bio3 | 0.205 | -0.152 | -0.372 | -0.022 | 0.278 |
| bio4 | -0.219 | 0.055 | 0.412 | 0.082 | -0.257 |
| bio5 | 0.198 | 0.367 | 0.241 | -0.011 | -0.108 |
| bio6 | 0.29 | 0.128 | -0.035 | 0.121 | 0.105 |
| bio7 | -0.25 | 0.136 | 0.263 | -0.186 | -0.249 |
| bio8 | 0.222 | 0.311 | 0.214 | -0.056 | 0.057 |
| bio9 | 0.28 | 0.163 | -0.053 | 0.098 | 0.087 |
| bio10 | 0.229 | 0.305 | 0.244 | 0.06 | -0.027 |
| bio11 | 0.282 | 0.188 | -0.051 | -0.008 | 0.126 |
| bio12 | 0.266 | -0.198 | 0.032 | -0.206 | -0.219 |
| bio13 | 0.257 | -0.06 | -0.149 | -0.297 | -0.353 |
| bio14 | 0.166 | -0.383 | 0.274 | 0.074 | 0.092 |
| bio15 | -0.135 | 0.268 | -0.423 | -0.21 | -0.093 |
| bio16 | 0.259 | -0.066 | -0.137 | -0.309 | -0.343 |
| bio17 | 0.176 | -0.38 | 0.266 | 0.071 | 0.057 |
| bio18 | 0.117 | -0.181 | 0.268 | -0.645 | 0.297 |
| bio19 | 0.21 | -0.181 | -0.088 | 0.269 | -0.579 |

| Supplementary table 4. Analysis of molecular variance (AMOVA) for *Plathymenia reticulata*. *F-statistics* which are significant (P < 0.05) are in bold. | | | | | | |  |
| --- | --- | --- | --- | --- | --- | --- | --- |
| Source of variation | d.f. | Sum of squared deviation | | Percentage of total variance | | F-statistics |  |
| *Localities* | | | | | | |  |
| Localities | 9 | 258.876 | | 18.76 | |  |  |
| Individuals within localities | 456 | 1126.35 | | 81.24 | | F_ST_ = **0.187** |  |
| Total | 465 | 1385.225 | |  | |  |  |
| *Ecotypes in all habitats* | | | | | | |  |
| Ecotypes | 1 | 251.135 | 22.17 | | F_CT_ **= 0.222** | | |
| Localities within ecotypes | 10 | 178.052 | 8.51 | | F_SC_ **= 0.109** | | |
| Individuals within localities | 454 | 1417.017 | 69.31 | | F_ST_ **= 0.307** | | |
| Total | 465 | 1846.204 |  | |  | | |
| *Ecotypes in Atlantic Forest and Cerrado (excluding ecotone)* | | | | | | |  |
| Ecotypes | 1 | 217.604 | 33.75 | | F_CT_ **= 0.337** | | |
| Localities within ecotypes | 4 | 56.417 | 5.78 | | F_SC_ **= 0.087** | | |
| Individuals within localities | 254 | 711.036 | 60.47 | | F_ST_ **= 0.396** | | |
| Total | 259 | 985.058 |  | |  | | |

Supplementary table 5: Matrix of pairwise F_ST_ between sampled localities of *Plathymenia reticulata*. All values are significantly different from zero ( P < 0.05).

| Localities | AJF | IPF | SJF | COE | NEE | FEE | SUE | PRS | PTS | VZS |
| --- | --- | --- | --- | --- | --- | --- | --- | --- | --- | --- |
| AJM | 0.000 |  |  |  |  |  |  |  |  |  |
| IPM | 0.072 | 0.000 |  |  |  |  |  |  |  |  |
| SJM | 0.088 | 0.099 | 0.000 |  |  |  |  |  |  |  |
| COE | 0.239 | 0.226 | 0.205 | 0.000 |  |  |  |  |  |  |
| NEE | 0.166 | 0.139 | 0.103 | 0.152 | 0.000 |  |  |  |  |  |
| FEE | 0.181 | 0.176 | 0.136 | 0.047 | 0.129 | 0.000 |  |  |  |  |
| SUE | 0.233 | 0.228 | 0.182 | 0.027 | 0.156 | 0.044 | 0.000 |  |  |  |
| PRS | 0.378 | 0.373 | 0.327 | 0.096 | 0.295 | 0.134 | 0.089 | 0.000 |  |  |
| PTS | 0.379 | 0.368 | 0.338 | 0.080 | 0.304 | 0.128 | 0.084 | 0.079 | 0.000 |  |
| VZS | 0.402 | 0.402 | 0.346 | 0.089 | 0.316 | 0.142 | 0.053 | 0.086 | 0.096 | 0.000 |

Supplementary table 6: Proportion of assignments in NewHybrids for the simulated data using different posterior probabilities thresholds (0.5, 0.75, 0.85, 0.95) based on pure *Plathymenia reticulata* populations for A) six simulated genotypic classes, B) 14 simulated genotypic classes assigned to six genotypic classes and C4) 14 simulated genotypic classes assigned to 12 genotypic classes. Only the non-zero values shown. P1=savanna ecotype, P2=forest ecotype, F1=savanna × forest, F2=F1 × F1, B1=F1 × savanna, B2= F1 x forest, B3=F2 x savanna, B4=F2 x forest, B5=savanna x (F1 x savanna), B6=forest x (F1 x forest), B7=savanna x (F2 x savanna), B8=forest x (F2 x forest), F3=F2xF2, F4=F3xF3.

| 1. Six hybrids simulated with 6 genotypic classes in NewHybrids | | | | | |
| --- | --- | --- | --- | --- | --- |
| Simulated Class | Classification | 0.5 | 0.75 | 0.85 | 0.95 |
| Forest | P2 | 0.940 | 0.930 | 0.900 | 0.870 |
| Forest | B1 | 0.060 | 0.020 | 0.010 | 0.000 |
| Savana | P1 | 0.980 | 0.930 | 0.910 | 0.840 |
| Savana | B2 | 0.020 | 0.010 | 0.010 | 0.000 |
| F1 | F1 | 0.950 | 0.850 | 0.790 | 0.560 |
| F1 | B2 | 0.010 | 0.010 | 0.000 | 0.000 |
| F1 | B1 | 0.020 | 0.000 | 0.000 | 0.000 |
| F2 | F1 | 0.030 | 0.020 | 0.020 | 0.010 |
| F2 | F2 | 0.610 | 0.460 | 0.390 | 0.280 |
| F2 | B2 | 0.140 | 0.100 | 0.060 | 0.010 |
| F2 | B1 | 0.170 | 0.100 | 0.060 | 0.020 |
| B1 | P2 | 0.070 | 0.050 | 0.030 | 0.020 |
| B1 | F1 | 0.030 | 0.030 | 0.030 | 0.010 |
| B1 | F2 | 0.030 | 0.010 | 0.010 | 0.000 |
| B1 | B1 | 0.860 | 0.730 | 0.660 | 0.300 |
| B2 | P1 | 0.060 | 0.020 | 0.020 | 0.020 |
| B2 | F1 | 0.030 | 0.020 | 0.020 | 0.010 |
| B2 | F2 | 0.030 | 0.000 | 0.000 | 0.000 |
| B2 | B2 | 0.870 | 0.750 | 0.650 | 0.390 |
| 1. 14 simulated classes with 6 genotypic classes in NewHybrids | | | | |  |
| Simulated Class | Classification | 0.5 | 0.75 | 0.85 | 0.95 |
| Forest | P2 | 0.990 | 0.960 | 0.960 | 0.780 |
| Forest | B2 | 0.010 | 0.000 | 0.000 | 0.000 |
| Savana | P1 | 0.980 | 0.960 | 0.960 | 0.820 |
| Savana | B2 | 0.020 | 0.000 | 0.000 | 0.000 |
| F1 | F1 | 0.890 | 0.600 | 0.600 | 0.090 |
| F1 | B1 | 0.030 | 0.000 | 0.000 | 0.000 |
| F2 | F1 | 0.010 | 0.010 | 0.010 | 0.000 |
| F2 | F2 | 0.670 | 0.510 | 0.510 | 0.190 |
| F2 | B2 | 0.150 | 0.050 | 0.050 | 0.030 |
| F2 | B1 | 0.140 | 0.040 | 0.040 | 0.020 |
| B2 | P2 | 0.050 | 0.010 | 0.010 | 0.000 |
| B2 | F1 | 0.020 | 0.000 | 0.000 | 0.000 |
| B2 | F2 | 0.010 | 0.000 | 0.000 | 0.000 |
| B2 | B1 | 0.920 | 0.790 | 0.790 | 0.340 |
| B1 | P1 | 0.020 | 0.010 | 0.010 | 0.000 |
| B1 | F1 | 0.010 | 0.000 | 0.000 | 0.000 |
| B1 | F2 | 0.010 | 0.000 | 0.000 | 0.000 |
| B1 | B2 | 0.930 | 0.850 | 0.850 | 0.260 |
| B4 | P2 | 0.030 | 0.020 | 0.020 | 0.000 |
| B4 | F1 | 0.020 | 0.000 | 0.000 | 0.000 |
| B4 | F2 | 0.030 | 0.000 | 0.000 | 0.000 |
| B4 | B1 | 0.900 | 0.750 | 0.750 | 0.300 |
| B3 | P1 | 0.060 | 0.010 | 0.010 | 0.000 |
| B3 | F1 | 0.010 | 0.000 | 0.000 | 0.000 |
| B3 | F2 | 0.030 | 0.010 | 0.010 | 0.000 |
| B3 | B2 | 0.880 | 0.740 | 0.740 | 0.260 |
| B3 | B1 | 0.010 | 0.000 | 0.000 | 0.000 |
| B6 | P2 | 0.390 | 0.290 | 0.290 | 0.140 |
| B6 | F2 | 0.010 | 0.000 | 0.000 | 0.000 |
| B6 | B1 | 0.600 | 0.540 | 0.540 | 0.240 |
| B8 | P2 | 0.400 | 0.330 | 0.330 | 0.090 |
| B8 | B1 | 0.600 | 0.500 | 0.500 | 0.270 |
| B5 | P1 | 0.400 | 0.350 | 0.350 | 0.140 |
| B5 | B2 | 0.590 | 0.540 | 0.540 | 0.250 |
| B7 | P1 | 0.460 | 0.410 | 0.410 | 0.200 |
| B7 | F2 | 0.010 | 0.000 | 0.000 | 0.000 |
| B7 | B2 | 0.530 | 0.460 | 0.460 | 0.080 |
| F3 | F2 | 0.670 | 0.480 | 0.480 | 0.190 |
| F3 | B2 | 0.100 | 0.070 | 0.070 | 0.000 |
| F3 | B1 | 0.150 | 0.070 | 0.070 | 0.020 |
| F4 | F2 | 0.700 | 0.470 | 0.470 | 0.220 |
| F4 | B2 | 0.130 | 0.080 | 0.080 | 0.000 |
| F4 | B1 | 0.140 | 0.090 | 0.090 | 0.030 |
| 14 simulated classes with 12 genotypic classes in NewHybrids | | | | |  |
| Simulated Class | Classification | 0.50 | 0.75 | 0.85 | 0.95 |
| forest | P2 | 0.820 | 0.490 | 0.130 | 0.000 |
| forest | B6 | 0.110 | 0.060 | 0.010 | 0.000 |
| savanna | P1 | 0.930 | 0.750 | 0.440 | 0.000 |
| savanna | B5 | 0.040 | 0.010 | 0.000 | 0.000 |
| F1 | F1 | 0.850 | 0.680 | 0.570 | 0.300 |
| F1 | F2 | 0.010 | 0.000 | 0.000 | 0.000 |
| F2 | F1 | 0.010 | 0.010 | 0.010 | 0.000 |
| F2 | F2 | 0.780 | 0.650 | 0.590 | 0.360 |
| F2 | B2 | 0.070 | 0.000 | 0.000 | 0.000 |
| F2 | B1 | 0.010 | 0.000 | 0.000 | 0.000 |
| F2 | B6 | 0.010 | 0.000 | 0.000 | 0.000 |
| B2 | F1 | 0.010 | 0.000 | 0.000 | 0.000 |
| B2 | F2 | 0.030 | 0.010 | 0.010 | 0.000 |
| B2 | B2 | 0.460 | 0.010 | 0.000 | 0.000 |
| B2 | B6 | 0.250 | 0.120 | 0.010 | 0.000 |
| B1 | F1 | 0.010 | 0.000 | 0.000 | 0.000 |
| B1 | F2 | 0.020 | 0.000 | 0.000 | 0.000 |
| B1 | B1 | 0.630 | 0.200 | 0.000 | 0.000 |
| B1 | B5 | 0.080 | 0.000 | 0.000 | 0.000 |
| B4 | F1 | 0.020 | 0.020 | 0.000 | 0.000 |
| B4 | F2 | 0.060 | 0.020 | 0.000 | 0.000 |
| B4 | B2 | 0.340 | 0.010 | 0.000 | 0.000 |
| B4 | B2 | 0.220 | 0.050 | 0.000 | 0.000 |
| B3 | F1 | 0.010 | 0.000 | 0.000 | 0.000 |
| B3 | F2 | 0.040 | 0.030 | 0.020 | 0.000 |
| B3 | B3 | 0.660 | 0.180 | 0.000 | 0.000 |
| B3 | B5 | 0.100 | 0.000 | 0.000 | 0.000 |
| B6 | P2 | 0.140 | 0.050 | 0.000 | 0.000 |
| B6 | F2 | 0.010 | 0.000 | 0.000 | 0.000 |
| B6 | B2 | 0.120 | 0.010 | 0.000 | 0.000 |
| B6 | B6 | 0.600 | 0.250 | 0.050 | 0.000 |
| B8 | P2 | 0.130 | 0.040 | 0.010 | 0.000 |
| B8 | B2 | 0.100 | 0.000 | 0.000 | 0.000 |
| B8 | B6 | 0.490 | 0.180 | 0.020 | 0.000 |
| B5 | P1 | 0.180 | 0.120 | 0.040 | 0.000 |
| B5 | B1 | 0.260 | 0.080 | 0.000 | 0.000 |
| B5 | B5 | 0.300 | 0.030 | 0.000 | 0.000 |
| B7 | P1 | 0.280 | 0.180 | 0.090 | 0.000 |
| B7 | F2 | 0.010 | 0.010 | 0.010 | 0.000 |
| B7 | B1 | 0.170 | 0.030 | 0.000 | 0.000 |
| B7 | B5 | 0.240 | 0.030 | 0.010 | 0.000 |
| F3 | F2 | 0.770 | 0.650 | 0.570 | 0.340 |
| F3 | B2 | 0.070 | 0.010 | 0.000 | 0.000 |
| F3 | B1 | 0.030 | 0.000 | 0.000 | 0.000 |
| F4 | F2 | 0.760 | 0.660 | 0.570 | 0.390 |
| F4 | B2 | 0.090 | 0.010 | 0.000 | 0.000 |
| F4 | B1 | 0.060 | 0.010 | 0.000 | 0.000 |
| F4 | B4 | 0.010 | 0.000 | 0.000 | 0.000 |

Supplementary table 7: Correlation among selected variables and dbRDA axes for *P. reticulata*. CEC= Cation exchange Capacity; CLIM4= Fourth bioclimatic component associated with Mean Diurnal Range of Temperature (bio2), Precipitation of Wettest Quarter (bio16) and Precipitation of Warmest Quarter (bio18); PCNM = second axis of the principal components of neighbour matrices representing a component of spatial distance between individuals.

| Variable | dbRDA1 | dbRDA2 | dbRDA3 |
| --- | --- | --- | --- |
| CEC | -0.958 | 0.278 | -0.066 |
| CLIM4 | -0.280 | 0.813 | 0.510 |
| PCNM2 | -0.816 | -0.034 | 0.578 |


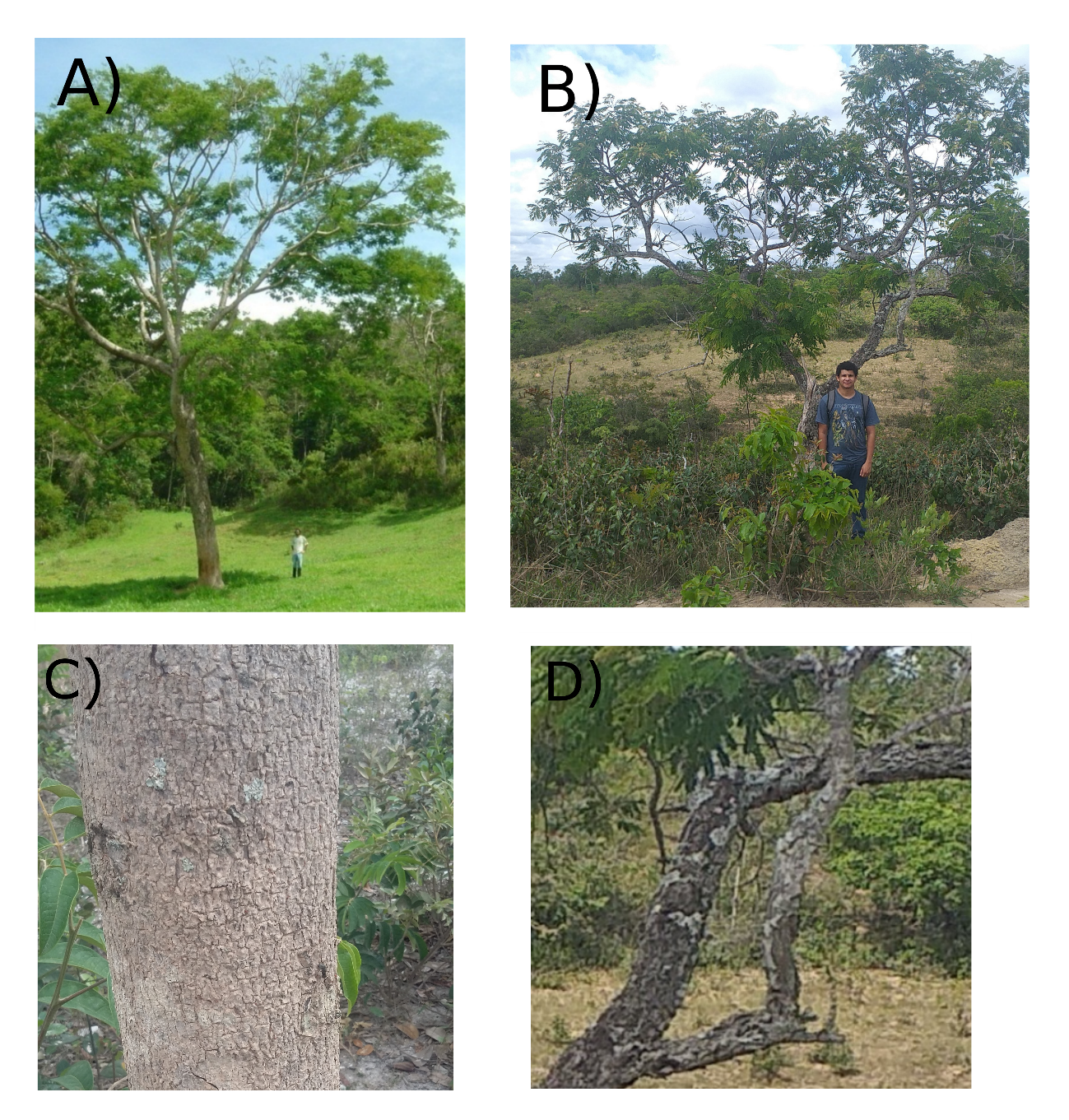


Figure S1: *Plathymenia reticulata* trees showing A) the straight trunk of forest ecotype, and B) the tortuous and twisted trunk of savanna ecotype. C) Trunk details of the forest ecotype and D) the suberous bark of the savanna ecotype.


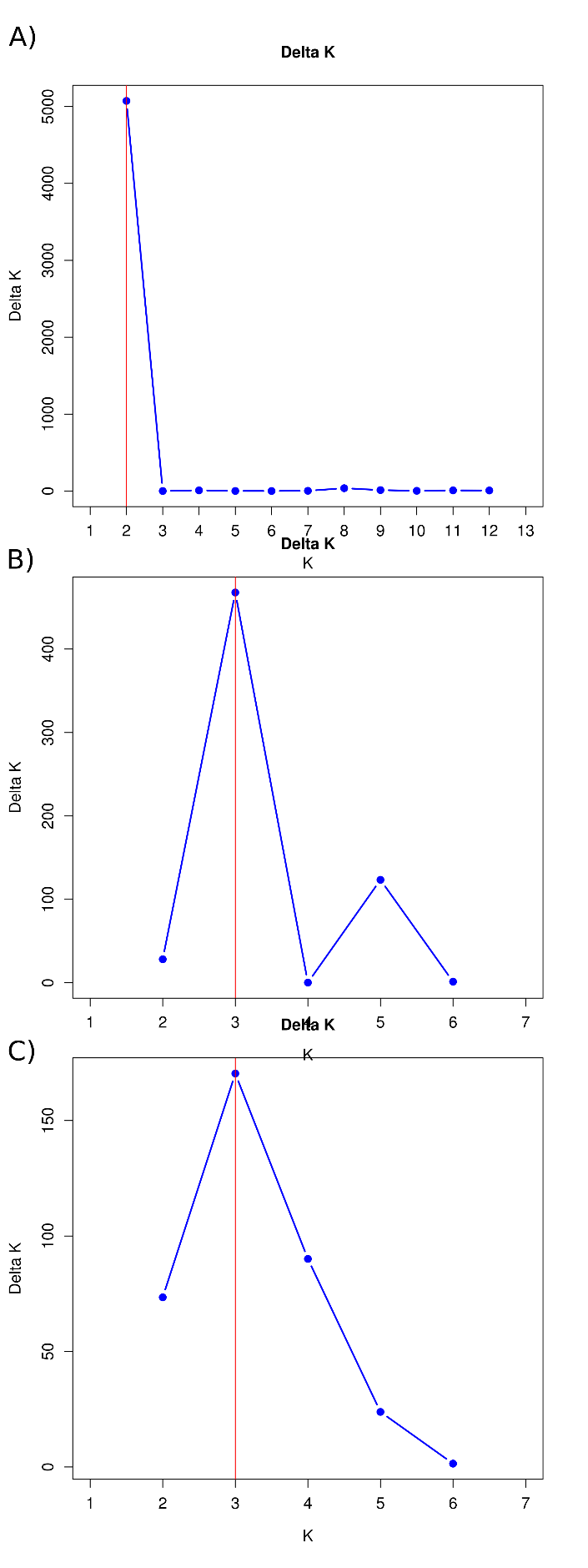


Figure S2: Evanno’s delta for sequential numbers of genetic clusters K evaluated in STRUCTURE software for A) all populations of *Plathymenia reticulata*, B) the pure individuals of forest ecotype and C) the pure individuals of the savanna ecotype of *P. reticulata*.


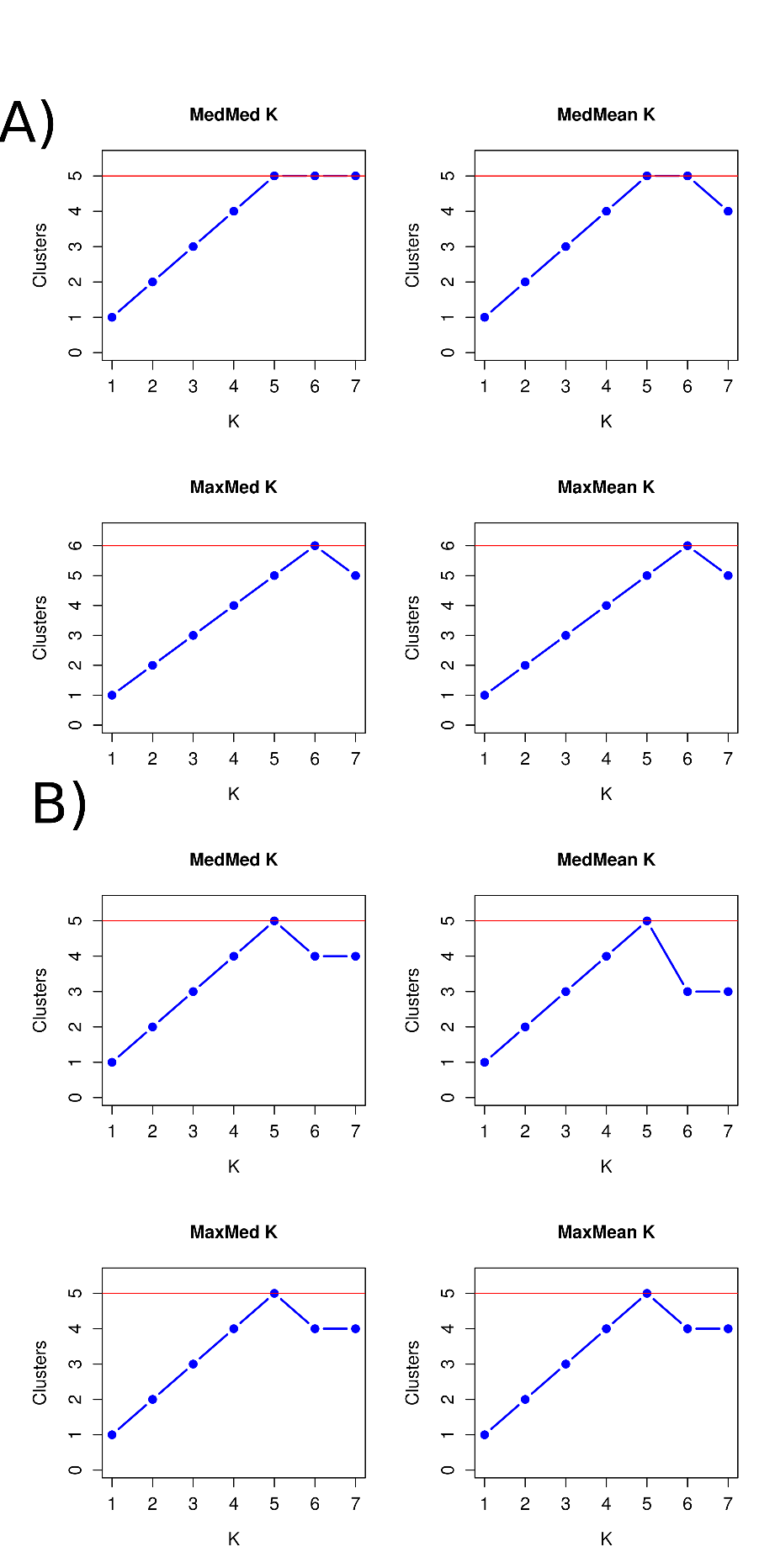


Figure S3: MedMeaK’ (median of means), ‘MaxMeaK’ (maximum of means), ‘MedMedK’ (median of medians) and ‘MaxMedK methods for chosen the best K in StructureSelector for A) the forest ecotype and B) the savanna ecotype of *Plathymenia reticulata*.


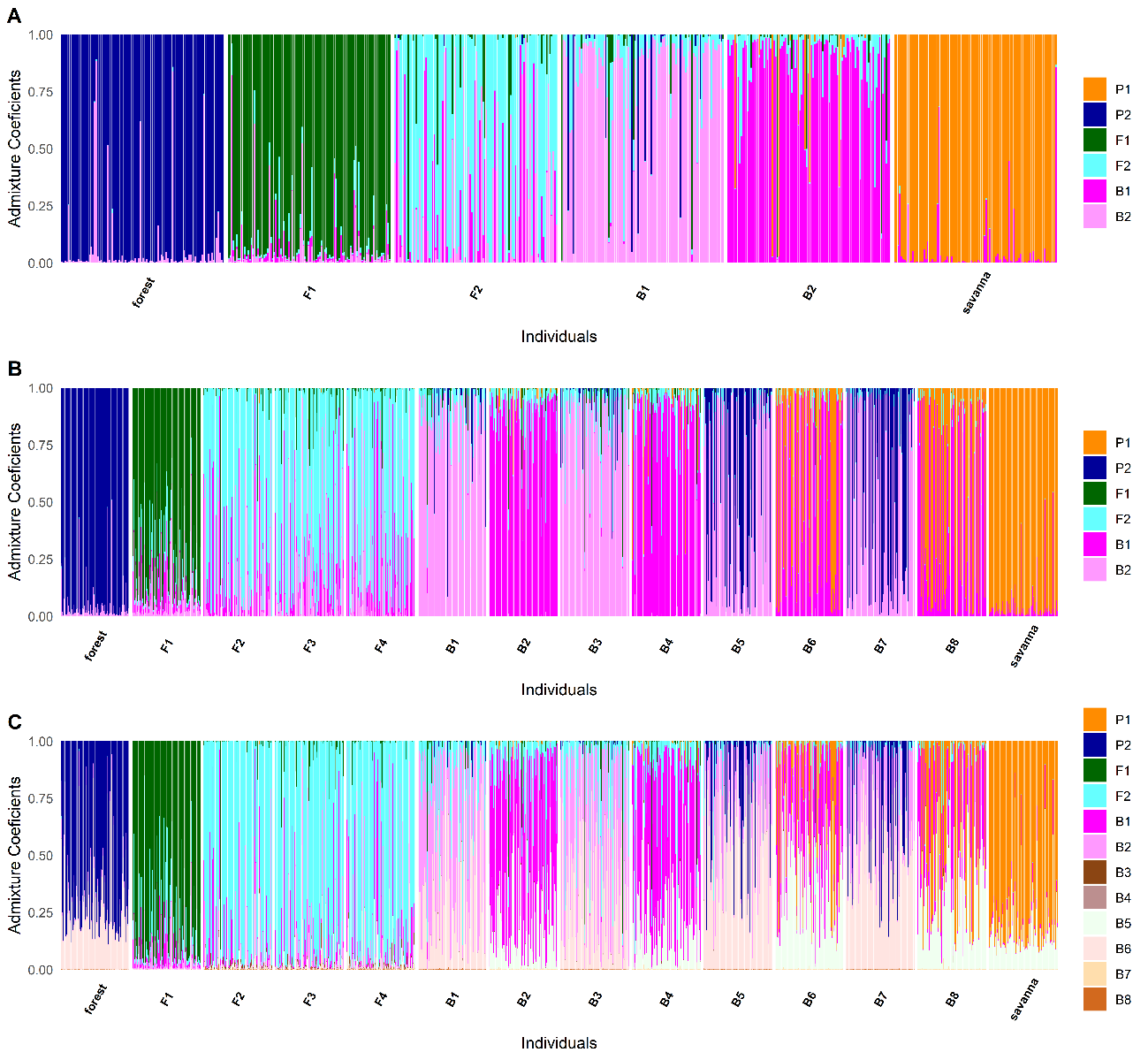


Figure S4: Barplots showing the posterior probability values estimated for each genotypic class to belong to assumed genotypic classes for each simulated individual in NewHybrids based on *Plathymenia reticulata* populations. A) Classification in six genotypic classes performed in simulated individuals of six simulated classes, two pure parental and four hybrid classes. B) Classification in six genotypic classes performed in simulated individuals of 14 simulated classes, two pure parental and 12 hybrid classes, being several later generation hybrids including first- and second-generation backcrosses and crossing between hybrids. C) Classification in 12 genotypic classes performed in simulated individuals of 14 simulated classes, two pure parental and 12 hybrid classes, being several later generation hybrids including first- and second-generation backcrosses and crossing between hybrids. P1=savanna ecotype, P2=forest ecotype, F1=savanna × forest, F2=F1 × F1, B1=F1 × savanna, B2= F1 x forest, B3=F2 x savanna, B4=F2 x forest, B5=savanna x (F1 x savanna), B6=forest x (F1 x forest), B7=savanna x (F2 x savanna), B8=forest x (F2 x forest), F3=F2xF2, F4=F3xF3.


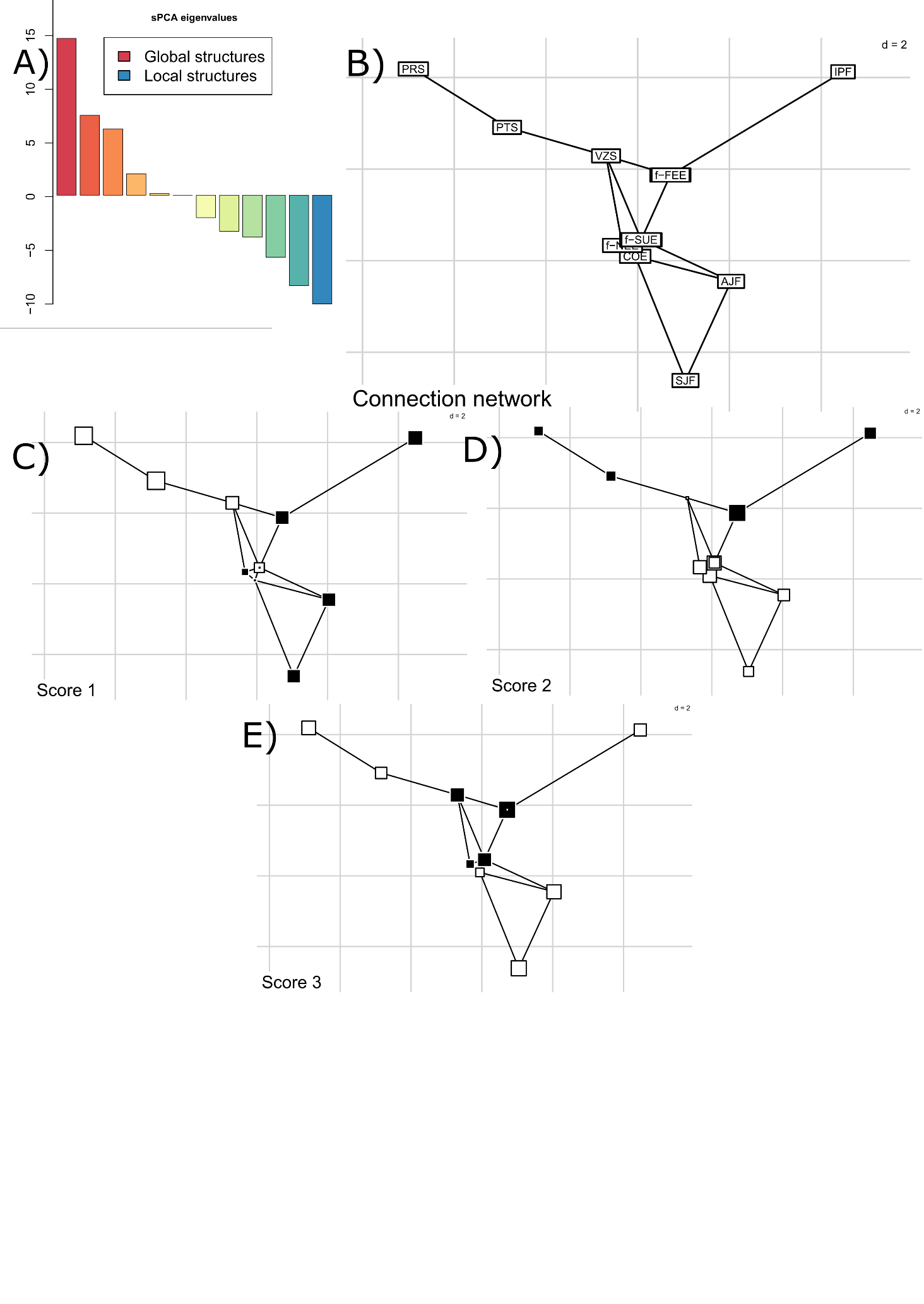


Figure S5: Spatial Principal Component Analysis (sPCA) showing A) the eigenvalues of each component of divergence, B) the sampling localities and the connection network among localities, and C) first, D) second and E) third positive axis of divergence in *P. reticulata* populations.

**Supplementary text 1: Evaluation of the performance of identification and classification of hybrids using STRUCTURE and *introgress***

The model evaluation in STRUCTURE based on simulated data (6 genotypic classes) showed a high accuracy in distinguishing admixed from non-admixed individuals, with a mean proportion of correct assignment of 99% and 95% for pure parentals and hybrids, respectively. However, the classification amongst different hybrid classes was poorer with a high similarity in admixture coefficients of F1 and F2, which is expected, but also with a few backcrossed individuals (Fig S6). Furthermore, the model evaluation showed an error rate of assignment of less than 5% for the simulated hybrids, with only a few backcrossed individuals being misclassified as pure parental populations (Fig S6). The data using the other later generation hybrids showed high accuracy with 100% of pure individuals of P1 and P2 showing admixture coefficients higher the 0.85 for pure individuals with several backcrosse classes being misclassified as pure individuals (Fig S6). In addition, the misclassification of backcrosses as pure individuals was 60% and 56% in some second-generation backcrosses (Fig S6). Finally, the crossing between hybrids (F1, F2, etc) were never classified as pure falling always in the interval of admixture coefficients between 0.150 and 0.850, with 66% of F1 showing values in this category (Fig S6). This indicates that the discrimination between pure individuals and later generation hybrids, specially, second-generation backcrosses is very poor.

The simulated data showed 96% and 97% of hybrid index values falling below 0.05 and above 0.95 for pure parental individuals, respectively (Fig S7A). However, 17% to 23% and 32 to 21% of the second-generation backcrosses with P1 and P2 showed values below 0.05 and above 0.95, respectively, indicating a poor capacity to discriminate between pure individuals and second-generation backcrosses. In addition, almost 40% of backcrosses fall into the “pure” category when the threshold was set to 0.85 and 0.15. 93% of the F1s hybrid index values fall between 0.4 and 0.6 and none F1, F2 and later hybrid crosses showed hybrid index values below 0.05 and 0.95 (Fig S7A). Only 42% and 41% of simulated pure individuals showed interspecific heterozygosity values below 0.1, with values in some later generation hybrids ranging from 0.01 to 0.10 (Fig S7B). 43% of simulated F2s, 46% of F3 and 32% of F4 showed values of interspecific heterozygosity above 0.5 while 96% of simulated F1s showed values above 0.5 with 0.36% of individuals showing interspecific heterozygosity values above 0.9 (Fig S7B). The mean values of Hybrid index x Interspecific heterozygosity show high similarity in these values for several simulated genotypic hybrid classes indicating difficult to discriminate among them (Fig S7C). For example, backcrosses of F1s and F2s with both parental showed very similar values indicating that they could not be discriminated correctly using this data. However, the mean value of Hybrid index x Interspecific heterozygosity for F1 and for parental were very different from most of other hybrid genotypic classes (Fig S7C).


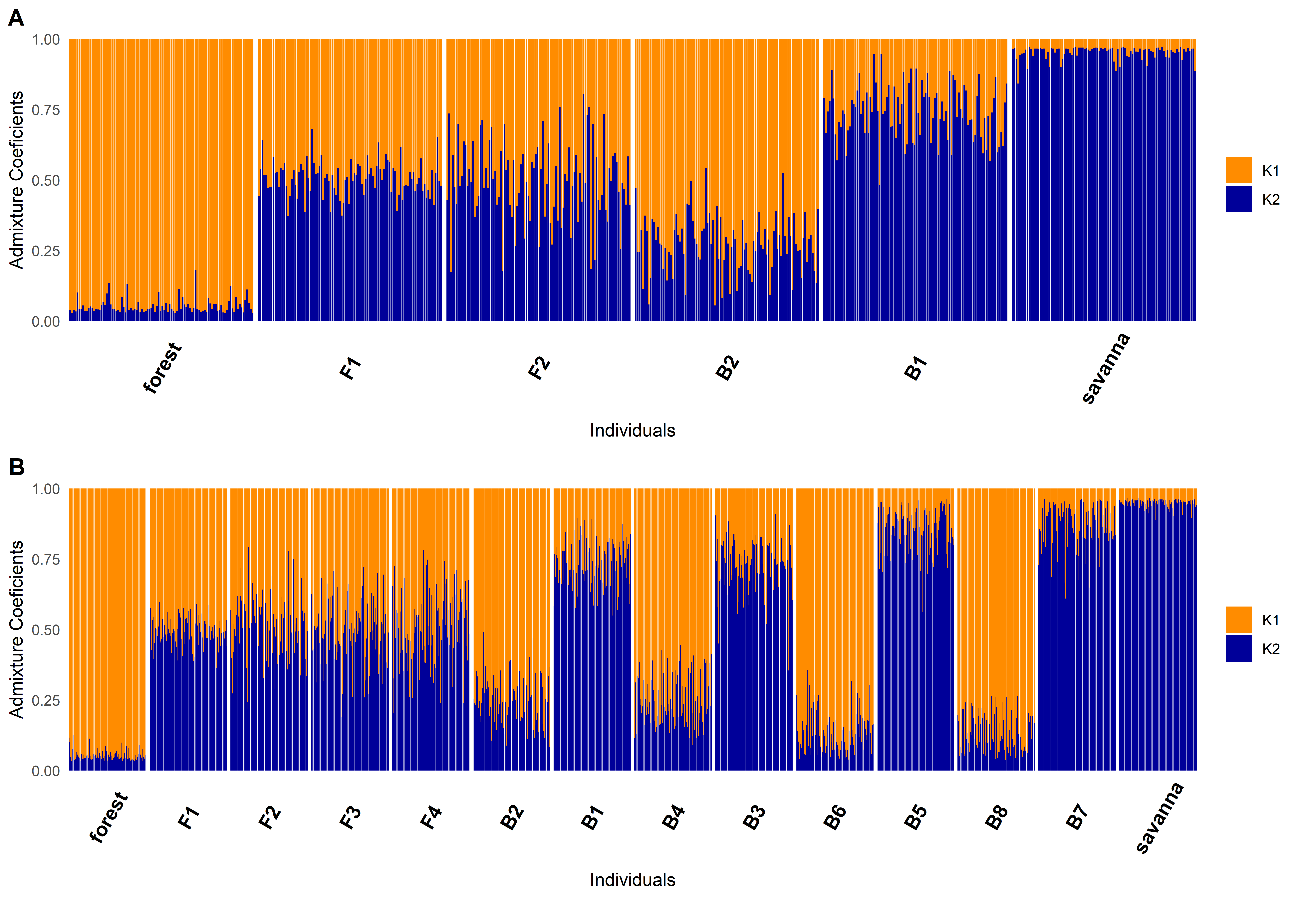


Figure S6: Box plots showing the observed distribution of admixture coefficients based on K = 2 estimated with the Bayesian clustering method STRUCTURE A) for *P. reticulata* populations and B) for the five simulated datasets for six genotypic classes. P1=savanna ecotype, P2=forest ecotype, F1=savanna × forest, F2=F1 × F1, B1=F1 × savanna, B2= F1 x forest, B3=F2 x savanna, B4=F2 x forest, B5=savanna x (F1 x savanna), B6=forest x (F1 x forest), B7=savanna x (F2 x savanna), B8=forest x (F2 x forest), F3=F2xF2, F4=F3xF2.


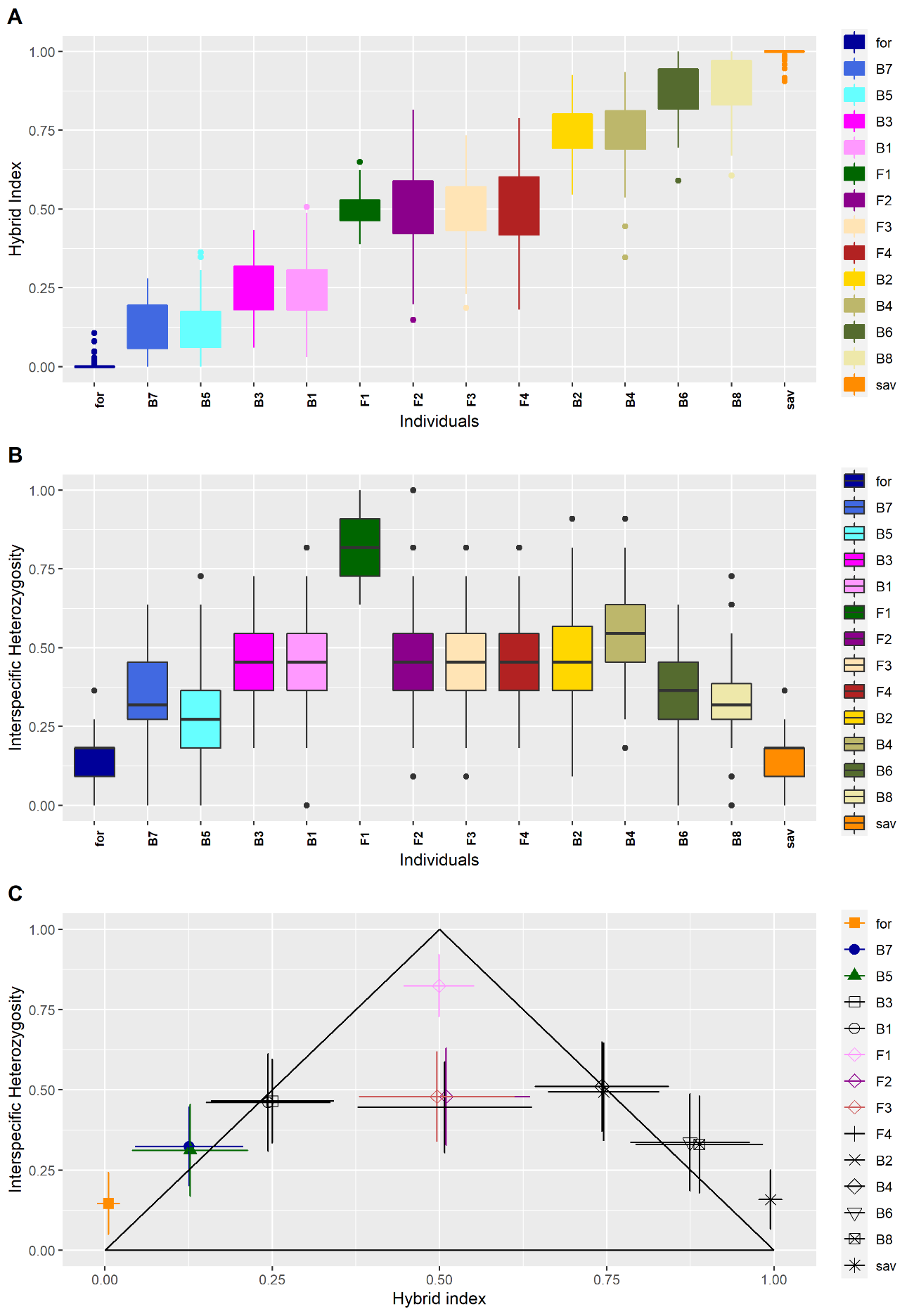


Figure S7: Evaluation of the performance of hybrid index and interspecific heterozygosity on simulated data in *Plathymenia reticulata*. (A) Box plots showing the distribution of index hybrid values estimated based on simulation using pure *Plathymenia reticulata* as parental. B) Box plots showing the distribution of interspecific heterozygosity values estimated based on simulation using pure *Plathymenia reticulata* as parental. C) Distribution of mean value and standard deviation for hybrid index and interspecific heterozygosity based on simulation using pure *Plathymenia reticulata*. P1=savanna ecotype, P2=forest ecotype, F1=savanna × forest, F2=F1 × F1, B1=F1 × savanna, B2= F1 x forest, B3=F2 x savanna, B4=F2 x forest, B5=savanna x (F1 x savanna), B6=forest x (F1 x forest), B7=savanna x (F2 x savanna), B8=forest x (F2 x forest), F3=F2xF2, F4=F3xF2.
